# Measuring affective well-being at work using short-form scales: Implications for affective structures and participant instructions

**DOI:** 10.1177/0018726717751034

**Published:** 2018-04-13

**Authors:** Emma Russell, Kevin Daniels

**Affiliations:** Kingston University, UK, e.russell@kingston.ac.uk; University of East Anglia, UK, kevin.daniels@uea.ac.uk

**Keywords:** affect, PANAS, positive and negative affect schedule, psychological well-being, psychometrics, short-form measures, validity

## Abstract

Measuring affective well-being in organizational studies has become increasingly widespread, given its association with key work-performance and other markers of organizational functioning. As such, researchers and policy-makers need to be confident that well-being measures are valid, reliable and robust. To reduce the burden on participants in applied settings, short-form measures of affective well-being are proving popular. However, these scales are seldom validated as standalone, comprehensive measures in their own right. In this article, we used a short-form measure of affective well-being with 10 items: the Daniels five-factor measure of affective well-being (D-FAW). In Study 1, across six applied sample groups (*N* = 2624), we found that the factor structure of the short-form D-FAW is robust when issued as a standalone measure, and that it should be scored differently depending on the participant instruction used. When participant instructions focus on *now* or *today*, then affect is best represented by five discrete emotion factors. When participant instructions focus on *the past week*, then affect is best represented by two or three mood-based factors. In Study 2 (*N* = 39), we found good construct convergent validity of short-form D-FAW with another widely used scale (PANAS). Implications for the measurement and structure of affect are discussed.

## Introduction

Psychological well-being at work has been well explored within the domain of organizational science as comprising affective, behavioural and cognitive components, such as positive and negative emotion, competence, integrative functioning and autonomy (Warr, 2003). Affective well-being (AWB) is considered to be the most important component of psychological well-being (van Horn et al., 2004; Warr, 1990), because of its proven relationship with many workplace constructs such as job satisfaction, job burnout, work–family conflict, occupational success and income (Hofmann et al., 2014; Ilies et al., 2015).

A key development in work and organizational research in recent years has been the increasing need to measure AWB with short scales. This appears to have been particularly promoted in two research contexts. Firstly, the emerging prevalence of repeated-measures, diary-based methodologies for examining fluctuations in AWB at an intra-individual level (Xanthopoulou et al., 2012) involves repeatedly asking people to assess their emotions over time. Secondly, given that AWB is such a significant predictor and outcome in organizational studies, measures of AWB are frequently administered as part of a suite of scales measuring individual and workplace constructs (McGonagle et al., 2015), whether in repeated-measures designs, independent-measures designs or cross-level designs.

In these contexts, researchers require measures of AWB to be short, to minimize disruption to normal tasks and memory (Fisher et al., 2016; Ohly et al., 2010), and to avoid irritating participants to the extent that response protocols are impacted (Hofmans et al., 2008; Robins et al., 2001; Stanton et al., 2002). This may be especially pertinent in field settings where the participant is also expected to undertake their usual work tasks (Russell et al., 2017). Capturing the causal and proximal influences on affective experiences in a quick, clear, time-bound manner (Miner et al., 2005; Ouweneel et al., 2012; Xanthopoulou et al., 2012) is also central to many current theoretical models that focus on people’s emotional experiences of work in an applied setting (including Affective Events Theory: Weiss and Cropanzano, 1996; Job-Demands Resources Theory: Bakker and Demerouti, 2007; and the Episodic Process Model: Beal et al., 2005). This is a research approach that has been especially pioneered by this journal (*Human Relations*, 2012, 65(9), Special Issue).

Despite the prominence of AWB as a concept across the psychological and behavioural sciences (Diener et al., 2015), measuring AWB with short scales across research contexts presents conceptual and psychometric challenges. In the current article, we identify and address these challenges by asking: does the use of short-form scales compromise the psychometric integrity and appropriate structural coverage of the AWB construct? To assess this, we examine two issues. Firstly, we aim to establish whether the structure of affect at work is represented differently when using a short-form measure, as compared with its long-form counterpart. In so doing, we utilize the Daniels’ (2000) five-factor measure of affective well-being (D-FAW), first presented and validated in its long form in this journal. With regard to the second issue, we aim to understand how short-form items used to measure AWB at work should be structurally represented when different time-bound focal instructions are used.^1^ For example, should discrete item scores be used (representing individual emotion terms) when assessing AWB momentarily, for example, ‘right now’, but aggregated item scores be used (representing mood constructs) when measuring AWB summatively, for example, ‘over the past week’? In the most part, momentary focal instructions are used in within-person designs, and summative focal instructions are used in between-person designs.

We make two key contributions with this research. Firstly, by examining whether shortening an AWB scale impacts on how people conceive of affect, we provide a focus that has been overlooked in the literature (Diener et al., 2010; Stanton et al., 2002). For although standalone short scales of AWB have been subjected to psychometric validation and reliability testing (Diener et al., 2010), the same rigour has not always been applied to standalone *short-form* scales, where a sub-set of items has been used from an existing scale, and where it is often assumed that the robustness of the long-form version necessarily transcends to a shortened scale (Cranford et al., 2006, is a notable exception). This appears to be a serious oversight as shortening measures of other constructs (such as the five factors of personality) reveals that reliability and construct validity can be compromised (Fisher et al., 2016; Hofmans et al., 2008). Secondly, and more broadly, we consider whether affect has a different structural representation (and therefore should be measured differently) when different time-bound focal instructions are applied in different contexts. This means that the scores extracted from a short-form AWB measure being used in a momentary assessment may be different from those extracted when using the same AWB measure in a summative assessment. These contributions are deemed especially important because of the extensive use of short-form AWB measures in occupational and organizational settings to inform current theory, research and policy development across the workforce and wider society (Diener et al., 2015).

### The structure and measurement of AWB

The structure and measurement of AWB has enjoyed healthy debate and research attention in recent years. Although some theorists assert that AWB is best represented by two independent dimensions of positive and negative affect that include terms at differing levels of activation (Tellegen et al., 1999; Watson and Clark, 1997), others argue for the superiority of a circumplex model, whereby specific terms can be differentiated by two orthogonal dimensions of hedonic tone and activation (Feldman Barrett and Russell, 1998; Larsen and Diener, 1992; Russell and Carroll, 1999). In both cases, affect is considered to comprise two components – one related to the hedonic tone or valence of the emotion (e.g. how positive or negative it is), and the other related to the activation or intensity of the emotion (e.g. whether it relates to a high or low arousal state) (Warr, 2003; Weiss and Cropanzano, 1996). To represent affect comprehensively, therefore, measures need to ensure that terms include a balance of positive and negative hedonic tone (valence), with different levels of activation. We do not enter the debate about how terms should be structurally arranged in this article. Rather, we assert that in shortening any AWB scale, the measure needs to maintain a balance of terms as outlined above. If this balance is lost, as a result of removing items, the shortened scale will be failing to provide full and balanced coverage of AWB as a construct.

**Figure 1.**
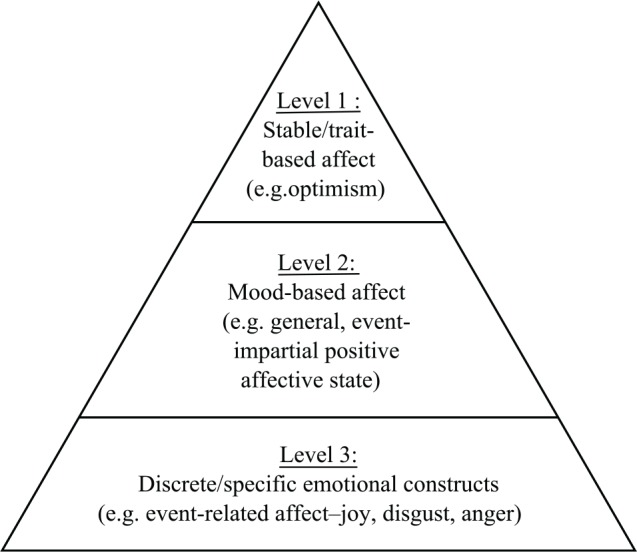
Different levels of affect.

In addition to the debate regarding the arrangement of affect terms, different levels of affect have been noted (see Figure 1). At the highest level (1), researchers focus on trait-based aspects of affect – relatively stable constructs that are highly correlated with (and possible sub-factors of) personality constructs such as Neuroticism and Extraversion (Beal and Ghandour, 2011; Costa and McCrae, 1980; DeNeve and Cooper, 1998; Steel et al., 2008). At the next level (2), mood-based aspects of affect include the experience of general positive or negative affective states (with high or low intensity/activation) that are not directly linked with any specific object or event (Brief and Weiss, 2002; Lazarus, 1991; Weiss and Cropanzano, 1996). The duration of the mood state can range from several days to several minutes (Weiss and Cropanzano, 1996). Finally, at the lowest level (3), affect is experienced in terms of discrete and specific emotional constructs, such as joy, disgust and anger, which are tied to an object or event in time (Frijda, 1993). Typically, affect at the lowest level is more likely to fluctuate and be experienced as a transient state (Xanthopoulou et al., 2012). An aggregation of base emotions (level 3) over time (and as the trigger object or event loses salience) provides a general mood experience (Frijda, 1993; Weiss and Cropanzano, 1996).^2^ To direct participants to the level of affect of interest, the focal instruction within the scale needs to indicate whether ratings are being made in the moment (e.g. ‘how you feel right now’), as a mood state (e.g. ‘how you felt today/this week’) or as a general indication of stable affect (e.g. ‘how you usually feel’).

### Measuring AWB in contemporary work-based designs

Traditionally, affect has been studied at the trait-based or mood-based level, and examined as a between-person construct (Beal and Ghandour, 2011; Miner et al., 2005). Taking one-off measures of AWB has meant that long-form scales have been suitable, and chosen because of the generally higher reliability and validity coefficients associated with longer measures (Watson and Clark, 1999). There are a number of well-established questionnaires available to assess AWB (Van Katwyk et al., 2000; Watson et al., 1988), but these are often too lengthy to use in contemporary organizational study designs that require brevity. Further, several established AWB measures have flaws in their psychometric construction, or are criticized for incompletely sampling affect (Diener et al., 2010; Feldman Barrett and Russell, 1998; Larsen and Diener, 1992; Van Katwyk et al., 2000).

For example, the positive and negative affect schedule (PANAS) is the most widely used measure of AWB (Diener et al., 2010; Kashdan et al., 2006). It was developed by Watson et al. (1988), but after criticism was levied that PANAS fails to measure low activation terms, and other essential affect components, it was updated to include a wider breadth of terms (Feldman Barrett and Russell, 1998; Larsen and Diener, 1992; Van Katwyk et al., 2000). However, this new version – the 60-item PANAS-X – takes about 10 minutes to complete (Watson and Clark, 1999), an example of concision being compromised for the sake of construct validity. Other well-being questionnaires have faced a similar dilemma – how to ensure that the measure comprehensively samples affect without being prohibitively long (Van Katwyk et al., 2000; Warr, 1990) – and are thus unsuitable for repeated-measures or multiple-scale data collection (Ohly et al., 2010; Ouweneel et al., 2012; Robins et al., 2001; Tschan et al., 2005).

Perhaps because of these issues, researchers assessing AWB in organizational studies frequently extract single items of affect or devise piecemeal short scales to measure the isolated constructs that they are interested in (Beal and Ghandour, 2011; Beal et al., 2006, 2013; Dockray et al., 2010; Elfering et al., 2005; Fisher et al., 2016; Ouweneel et al., 2012; Parkinson et al., 2016), without necessarily considering how this approach impacts on measurement protocols or whether items reflect the valence/activation balance of underlying affect structures. Such an approach is not seen in other fields, and has been advised against by measurement theorists (Boyle, 1991; Kline, 1986; Stanton et al., 2002). For example, in measuring personality in the short form, researchers do not extract single-factor scales from the five-factor model (FFM), as this would remove essential controls (Bernerth and Aguinis, 2016). Rather, standalone short-form scales of the full FFM are utilized, and much research has been conducted to assess how reliable and comprehensive these shortened personality scales are (Cooper et al., 2010; Gosling et al., 2003; Hofmans et al., 2008).

Further, it is usual in affect measurement to apply different focal instructions to scales. Yet, in line with theories about the structure of affect (see Figure 1), assessing whether the item scores should then be summed to form a composite, or analysed as individual items, is not always considered. For example, in momentary affect measurement, a mood-based aggregate score (e.g. NA or PA) is sometimes used (Dimotakis et al., 2011; Elfering et al., 2005); at other times, scores for discrete items are reported (Beal et al., 2013). With regard to the measurement of AWB in organizational studies, we identify two key elements that require evaluation: (a) whether the standalone short-form version of a validated long-form measure of AWB still captures the construct comprehensively when long-form items have been removed; and (b) whether the time-bound focal instruction, used in measures of AWB, reveals differences in the underlying structure of affect that will impact how the short-form measure should be scored in different contexts. In *short-form* measures, we refer to standalone questionnaires whereby all of the items are found in a long-form version but the short-form version of the questionnaire is administered as a standalone scale in its own right (see Appendix 1, for example).^3^ These elements are now explored below.

#### Issues with shortening a long-form scale

In general, short-form scales are subject to reduced levels of internal consistency (Romero et al., 2012) or construct validity (Stanton et al., 2002). Where scales are bipolar, shortening a measure can cause psychometric problems. Providing just two terms or items per scale, that are opposite to each other in meaning, is beneficial in terms of capturing the facet more completely (Gosling et al., 2003). However, it does mean that internal consistency suffers (Cooper et al., 2010; Romero et al., 2012; Stanton et al., 2002). Indeed, traditional analyses of reliability (Cronbach’s alpha) with very short (e.g. two items) bipolar scales are not recommended as a sound method for assessing whether scales have construct integrity (Geldhof et al., 2014; Parkinson et al., 2016; Rush and Hofer, 2014; Stanton et al., 2002; Woods and Hampson, 2005). Alternative approaches, such as the use of test–retest reliability, can be helpful in measuring static constructs (Gosling et al., 2003; Robins et al., 2001; Woods and Hampson, 2005) and are used in place of Cronbach’s alpha to establish reliability (Rammstedt and John, 2007) in short scales. However, this makes little sense when measuring constructs that we expect to fluctuate (Fisher et al., 2016), such as AWB. Indeed, Boyle (1991) indicates that in shortening scales designed to measure mood or affect, priority should be placed on capturing the construct effectively, even if this is at the expense of establishing acceptable Cronbach’s alpha statistics. The recommendation, then, is that assessing the fit of factor structures (associated with long-form scales or theoretical constructs) should be used as the best means of establishing construct integrity in short scales (see Boyle, 1991; Kline, 1986; Rammstedt and John, 2007; or Stanton et al., 2002, for a discussion).

In a short-form measure, where items from the long form have been removed, we argue that the removal of emotion terms should be balanced across valence and activation components. Yet, even when a standalone short-form scale contains items that maintain representation of the complete AWB construct, the loss of items can alter the rating context. For example, when multiple terms are used for each sub-scale in a long-form measure, participants may cognitively group items, which is then reflected in the factor structure. With fewer items in a standalone short-form measure, the participant may consider each item separately, or group items in a different way, allowing factors to emerge that may not replicate the original factor structure of long-form versions of the questionnaire (Cooper et al., 2010). Indeed, in a study of a balanced and construct-valid standalone short-form personality measure, two out of five factors failed to replicate the long-form structure, requiring the authors to alter the short-form terms to improve dimensional representation (Hofmans et al., 2008).

In measuring AWB in the short form, then even a representative balance of items may potentially imply a factor structure to the organization of affect that differs from the long form. It must be noted that in the present studies we are not interested in comparing a long-form measure of AWB with its short-form equivalent *in the same sample*. It would be untenable to administer long- and short-form versions of a scale to participants at the same time because of the replication of all short-form items in the long form. Rather, the present studies are concerned with comparing the factor structure of a standalone short-form AWB measure with the factor structure of its long-form version, and identifying whether the short form is still construct valid and reliable.

#### Issues with using different focal instructions

The focal instruction of a measure is designed to direct the rater’s attention to the temporal and contextual boundaries of the construct being rated. For example, focal instructions that ask participants to consider how often they generally experience their moods or emotions are tapping into higher constructs (Figure 1: level 1) at a trait level (Watson et al., 1988). Asking how one generally felt over a particular timeframe (e.g. the past day or week) accesses summative affect (Figure 1: level 2), such as mood (Beal and Ghandour, 2011; Diener et al., 2010). Using a focal instruction that asks how one feels ‘right now’ at a moment in time is accessing momentary AWB (Figure 1: level 3), especially when specific (afraid or angry) rather than summary (bad or negative) terms are used (Diener et al., 2010).

Changing the focal instruction can impact ratings of affect, as it provides the anchor to which affect is positioned (Xanthopoulou et al., 2012). For example, Weiss and Cropanzano (1996) suggest that in measuring emotion as a current state, the hedonic tone of the emotion is especially salient to the rater, because they are focused on pleasurable feelings in relation to a specific event. Warr (1990) also suggests that people tend to focus on the ‘good’ or ‘bad’ of their feelings when measuring affect across shorter timeframes. Examining whether and how focal instructions might alter ratings of affect is therefore a current concern.

### The present studies

In the present studies, our focus is on examining whether the 10-item D-FAW, as a short-form measure of AWB at work, can adequately and completely capture the construct of affect, as represented by 30-item D-FAW, without compromising psychometric integrity as a result of either the brevity of the measure (Robins et al., 2001; Russell, Weiss and Mendelsohn, 1989), and/or the time-bound guidance (momentary and summative) of the measure’s focal instruction (Diener et al., 2010).

In order to address our research question, we need to use a measure of affective well-being that: (a) provides terms that represent the structure of affect across terms reflecting different levels of activation and positive and negative valence; (b) maintains this breadth of construct representation in both its long- and short-form versions; (c) has demonstrated criterion-related validity in the short form, providing confidence that it is a tool that has utility and application in organizational contexts; and (d) provides a flexible focal instruction to allow for the momentary and summative assessment of affect.

Daniels’ (2000) five-factor model of affective well-being (D-FAW) was originally devised as a 30-item scale but has been shortened to 10 items for use in organization studies (Daniels and Harris, 2005; Harris and Daniels, 2005; Russell et al., 2017). Figure 2 (available online as supplementary material) represents how the 10-item measure would map onto the best-fitting factor structure of the long-form D-FAW (also outlined below). The 30-item measure is an extension of a two-factor measure developed by Warr (1990) and has been validated and assessed for the best-fitting factor structure across a range of work contexts (Daniels, 2000). The five factors are anxiety–comfort (AC), angry–placid (AP), displeasure–pleasure (DP), tiredness–vigour (TV) and bored–enthusiastic (BE). The five factors of D-FAW can be mapped onto the second-order solution of Positive Activated Affect (PA) and Negative Activated Affect (NA) (Feldman Barrett and Russell, 1998). AC and AP load onto NA, and TV and BE load onto PA. DP can be a stand alone factor, or can load onto either PA or NA (see Daniels, 2000, for a discussion). The 10-item short form attempts to maintain the construct representation of the long-form version, with the five factors each containing one positively and one negatively valenced term, balanced in activation. The breadth of discrete factors, representing the broad structure of affect, and the availability of a shorter – but equivalently balanced – 10-item form, means that the D-FAW is suitable for addressing our research question. Further, in its short form, the D-FAW has been used to identify relationships between job stressors, demands, goal progress and beliefs (Daniels and Harris, 2005; Harris and Daniels, 2005; Harris et al., 2003; Russell et al., 2017) across a range of occupational contexts. As such, the criterion-related validity of the short-form D-FAW does not require elucidation in this article. Finally, D-FAW allows researchers to alter the focal instruction in order to focus raters’ attention on the timeframe of affect or job context appropriate to the application. This means that affect at any of the levels of the affect hierarchy (see Figure 1) can be captured.

Two studies were conducted to establish whether D-FAW retains its long-form factorial structure, construct validity and psychometric integrity when used to collect momentary and summative AWB data in the workplace. In the first study, data were used from six samples that had previously been administered the D-FAW in applied organizational studies. References to the original study and purpose are outlined in Table 1. Full permissions to use the data from these studies were obtained prior to the analysis in the present study. For each sample, data were modelled according to different affect factor structures, originally tested in the long-form version (Daniels, 2000). In the second study, we examine whether shortening a scale affects the construct validity of the measure. A construct validity analysis of the 10-item D-FAW, using momentary focal instructions, was performed with the 20-item PANAS (Watson et al., 1988). The PANAS was chosen as the comparison measure for the validity study because of its widespread popularity (Diener et al., 2010; Kashdan et al., 2006). However, it is acknowledged that the 20-item PANAS is not an occupationally specific measure, lacks coverage of low activation items (Diener et al., 2010; Tellegen et al., 1999) and has low representation of anger/aggression terms (Daniels, 2000) and fatigue (Watson and Clark, 1997).

**Table 1.** A summary of the samples used in Study 1.

	Sample 1	Sample 2	Sample 3	Sample 4	Sample 5	Sample 6
Gender	M = 150; F = 122	M = 580; F = 1200	M = 133; F = 272	M = 5; F = 31	M = 26; F = 13	M = 40; F = 66
Age	Mean 42.0 (SD = 10.4)	Mode 46–50 (21.3%)	Mode = 31–40 (35%)	Mean = 34.0	Mean = 25.9 years	Mode = 21–30 (43%)
Industry sector	Manufacturing and local government	University workers	Utilities (energy), journalism and publishing, charity sector, insurance, airline, consultancy, university sector	Human resource department in a hospital	Graduates at multinational blue-chip technology firm	Finance and accountancy, architecture, media, insurance, charity sector, university sector
Purpose (and reference) of original study	Examining beliefs about stressors’ relationships with a range of variables including AWB (Daniels et al., 2002)	Validation of Daniels’ (2000) scales (Harris and Daniels, 2005)	Examining how strategies for dealing with email relate to well-being ratings (Russell, 2013)	Examining how beliefs about stressors relate to affective well-being (Harris and Daniels, 2005)	Examining convergent validity of 20-item PANAS with 10-item D-FAW (sample unique to this article)	Examining AWB after dealing with naturally occurring email interruptions (part inclusion in Russell et al., 2017)
Number of participants (*N*)	244	1794	405 (340 after missing data)	36	39	106
Number of cases (multilevel only) (*n*)	N/A	N/A	N/A	284	567 (3 times daily for 10 days; some missing data)	965 (after dealing with each email interruption over one study period)
Number of items (D-FAW)	10	10 (extracted from 30-item)	10	10	10	10
Sampling method	Entire workforce of selected departments (response rate 38%)	Entire workforce of organization (response rate 58%)	Opportunity (response rate: unavailable)	Opportunity (response rate: 77%)	Opportunity (response rate: 85% from original agreement to participate)	Opportunity (response rate: 79% from original agreement to participate)
Focal instruction (How you feel/felt …)	Over the past week	Over the past week	Over the past week	Today	Right now, at the present moment	Right now, at the present moment
Affect Focus	Summative	Summative	Summative	Summative	Momentary	Momentary (event-specific)

M = male; F = female; SD = standard deviation; N/A = not available; D-FAW = Daniels five-factor measure of affective well-being; PANAS = positive and negative affect schedule; AWB = affective well-being.

## Study 1

In Study 1, the factor structure of the 10-item D-FAW, using different introductory focal instructions across six samples, was analysed. Details about the samples are provided in Table 1. It was anticipated that in the short-form measure of D-FAW, affect might be structured differently according to whether the focal instruction focused on either momentary (‘right here, that is at the present moment’) or summative (I felt this way ‘today’/‘last week’) timeframes. All samples (bar Sample 2) only completed the 10-item D-FAW (see Appendix 1). Sample 2 completed the 30-item long-form D-FAW, but we then extracted the short-form 10-items from this (see Appendix 2, available online as supplementary material). Samples 1–3 completed D-FAW with a summative focal instruction in a between-persons design. Sample 4 completed D-FAW with a summative focal instruction in a within-persons design, and Samples 5 and 6 completing D-FAW with a momentary focal instruction in a within-persons design.

Fourteen different models were fitted, based on how affect may differently be arranged at different levels, reported each time as a model first without and then with response bias factors controlled for. Only models that represent established models of affect were tested, along with other factor structures that were tested in the validation of the original long-form 30-item measure of D-FAW (Daniels, 2000). These models represent: (a) a one-factor model, representing an overall single well-being factor (Berkman, 1971; Cropanzano et al., 2003; Wright and Staw, 1999) (Models 1 and 2, see Figure 2a, available online as supplementary material); (b) a two-factor model consisting of NA and PA factors, representing the Watson and Tellegen (1985) or Feldman Barrett and Russell (1998) approach (Models 3 and 4, see Figure 2b, available online as supplementary material); and (c) a five-factor model representing Daniels’ (2000) five-factor model (Models 5 and 6, see Figure 2c, available online as supplementary material).

Next: (d) Models 7 and 8 (see Figure 2d, available online as supplementary material) represent a hybrid of Models 5 and 6 (first-order five factors) with Models 1 and 2 (second-order one factor); (e) Models 9 and 10 (see Figure 2e, available online as supplementary material) fit discrete first-order factors for PA (BE, TV and DP) with a single NA factor. Then, (f) Models 11 and 12 (see Figure 2f, available online as supplementary material) fit discrete first-order factors for NA (AC, AP and DP) with a single PA factor. Finally, (g) Models 13 and 14 (see Figure 2g, available online as supplementary material) represent the best-fitting structure for the long-form D-FAW, with five first-order factors loading onto two second-order factors of NA and PA.

In response to a request from an anonymous reviewer, we also tested a model containing two ‘response bias’ factors – with factor 1 representing only positively valenced items and factor 2 representing only negatively valenced items. The results of this testing are presented in Appendix 4 (available online as supplementary material).

### Response bias

When rating constructs that differ in terms of their favourableness, response bias can become an issue. In measuring AWB, response biases are usually directed towards responding favourably to questions about feeling positive (e.g. happy, contented, joyful), and people are less willing to admit to feeling angry, lonely or tormented (Gotlib and Meyer, 1986). Scales that only contain positively valenced items therefore not only fail to represent the structure of most models of affect, but are also likely to succumb to response bias when rated. Because response bias issues may obscure the true nature of well-being (Warr, 1990), it is suggested that response bias factors for positive and negative worded items are included in assessments of factor structures (Gotlib and Meyer, 1986). This is why each of the models that we ran was tested with and without response bias factors.

Based on the theoretical reasoning presented in Figure 1, we generate our first two hypotheses. Measuring affect as a momentary construct involves assessing emotions as discrete and individual event-bound constructs. The long-form version of D-FAW revealed that a five-factor first-order solution of discrete bipolar scales, loading onto second-order factors of NA and PA was the best fit for the data (Daniels, 2000; Model 14 in the present article). We expect that the best fit for discrete momentary assessments will represent the five factors of the original D-FAW (Model 6). Whereas, when affect is rated as a summative construct, generalizations are made about mood that allow for positive and negative, activated and non-activated emotions to be present within the same timeframe (Weiss and Cropanzano, 1996), and a two-factor (PA and NA) solution (Model 4) would be a better fit (Watson and Tellegen, 1985).

*Hypothesis 1*: The best-fitting factor structure for the 10-item D-FAW using a momentary focal instruction is a discrete first-order five-factor solution (Model 6).
*Hypothesis 2*: The best-fitting factor structure for the 10-item D-FAW using a summative focal instruction is a first-order two-factor PA and NA solution (Model 4).

Sample 2 uses a summative focal instruction, along with samples 1, 3 and 4. However, Sample 2 was administered as 30 items, with 10 items subsequently analysed (whereas samples 1, 3 and 4 used 10-item standalone short-form administrations – see Appendices 1 and 2). The two-tailed hypothesis offered for Hypothesis 3 is based on the supposition that the context of the other 20 emotion terms will impact on how the extracted 10-item D-FAW terms are rated, but we cannot predict how this will affect the factor structure.

*Hypothesis 3*: The best-fitting factor structure for Sample 2 will be different from the solution best-fitting Samples 1, 3 and 4.

### Method

#### Sample

Six samples were used in this analysis. Table 1 includes a summary of the demographics of each sample, the focal instruction used by participants in taking the D-FAW in each sample, the sampling method, and details of the version length of the D-FAW used for each cohort. As mentioned, each of the samples took the D-FAW when participating in different studies investigating the relationship between AWB and another construct. Details are also provided in Table 1.

#### Materials and procedure

The 10-item version of D-FAW (Harris and Daniels, 2005) presented the following terms, with the primary factor loadings and scoring direction (+ or –) shown in brackets: Happy (DP+), At ease (AC–), Anxious (AC+) Annoyed^4^ (AP+), Motivated (TV+), Calm (AP–), Tired (TV–), Bored (BE–), Gloomy (DP–^5^), Active (BE+). The 30-item long-form measure was used for Sample 2 only, and uses the same terms as outlined in Daniels’ (2000) article. Ten items were then extracted for analysis from the 30-item measure, as per the 10 terms described above (see Appendix 2, available online as supplementary material). There were three different focal instructions. Summative AWB was measured in Samples 1–3, using ‘Thinking of the past week, how much of the time has your job made you feel each of the following …?’ In Sample 4, summative AWB was measured using, ‘How work has made you feel today’ (rating each item). Momentary AWB (Samples 5 and 6) was measured using, ‘Indicate to what extent you feel this way *right now, that is, at the present moment* ….’ Items were scored on 1–6 response rating scales, where 1 = not at all and 6 = very much (the extent that the given adjective describes how they feel) in all samples. In Samples 4–6, measures of AWB were collected on a repeated-measures basis, and therefore the structure of the data for these samples is multilevel (AWB at each time point represents level-1, nested within the person at level-2). Participants completed the D-FAW either on-line (Sample 3), or using a pen or pencil – either at designated periods (Sample 1, 2, 4 and 5) or after responding to an event (Sample 6).

#### Analysis

Fourteen possible models were tested for each sample. All models were analysed in M-Plus (Muthén and Muthén, 1998), using Bayesian Confirmatory Factor Analysis (B-CFA). Multilevel B-CFA was required for Samples 4, 5 and 6, which separates out the within- and between-person variance, but enables better estimation when the number of level-2 participants is low and/or the models are complex (Muthén, 2010). For comparability purposes, data across all samples were analysed using multilevel B-CFA.

Parsimony or fit was assessed for each model by using three indices (Muthén, 2010): potential scale reduction (PSR), where a level less than 1.1 indicates convergence, but not necessarily fit; posterior predictive checking (PPC), which should ideally be non-significant (*p* > .05), to indicate fit; deviance information criterion (DIC) indicates a better fit the smaller it is compared with other models. These indices can cope with reliability values that are below acceptable limits (Browne et al., 2002) because they allow comparisons across samples for best-fitting models.

For all models, one of the indicators for each factor was fixed to 1 (as in standard CFA). Substantive affect factors were usually allowed to correlate (e.g. NA with PA). First-order affect factors that loaded on a second-order factor were not allowed to correlate with other affect factors. Response bias factors were set to be orthogonal to affect factors, but were allowed to correlate with each other. Each model was also specified with informative but wide-banded priors for factor loadings (i.e. items loading in the expected direction) to aid estimation. For substantive first- and second-order affect factors (e.g. NA), priors were set to 1 with a standard deviation of .40. This implies that 95% of loadings should fall between .20 and 1.80. The lower bound is below the conventional threshold for an acceptable loading in exploratory factor analysis (i.e. .30). In models with response bias factors, priors for response bias factor loadings were set to 1 with a standard deviation of .65 (implying 95% of loadings should fall within the range –.30 to 2.30).

### Results

Descriptive statistics are reported in Table 2 (available online as supplementary material) for each sample. The results of the descriptive statistics indicate that, when scored in the positive direction, participants generally rate their well-being as being above the mid-point on each scale. TV is the scale that shows the lowest overall mean ratings across the samples. Generally, the skew and kurtosis values are within the +/–1 standard error boundaries of acceptability (Field, 2009) across all samples. The exception is for Sample 4, where BE and DP are negatively skewed (–1.11 and –1.16, respectively) and leptokurtic (2.76 and 1.45, respectively). DP in Sample 5 is also just beyond the limit of acceptability for skewness, indicating a slight negative distribution (–1.04).

Results of the B-CFA are presented in Table 3 (available online as supplementary material). PSR, PPC and DIC statistics are presented, along with an indication as to whether loadings were in the hypothesized direction or not. Taken together, these figures allow us to establish which models provide the best fit for the data. As seen in Table 3 (available online as supplementary material), the inclusion of response bias factors tended to improve model fit. Therefore, it appears to be necessary to have a balance of positively and negatively valenced items in order to eliminate confounding owing to response biases. In the remainder of this section, we focus only on models with response bias factors included.

#### Best-fit model for momentary focal instructions

Samples 5 and 6 contained momentary focal instructions for the 10-item D-FAW. The two best-fitting models for Samples 5 and 6 were Model 6 (best fit in Sample 5, second best fit in Sample 6) and Model 12 (second best fit in Sample 5, best fit in Sample 6). However, not all items in Model 12 loaded significantly on the assigned factor in either sample, but all items loaded significantly and in the hypothesized direction in both samples for Model 6. Therefore, the results support Hypothesis 1.

#### Best-fit model for summative focal instructions

Samples 1, 3 and 4 used summative focal instructions (Samples 1 and 3 used ‘past week’ and Sample 4 used ‘today’) for the 10-item D-FAW. Sample 2 also used a summative focal instruction (‘past week’), but this was for the 30-item D-FAW with 10 items extracted. The best-fitting models for using ‘summative’ focal instructions on the 10-item D-FAW (Samples 1, 3 and 4) were Models 6 and 12, in that the DIC was lowest across these samples *and* the loadings were more likely to be significant in the hypothesized direction. Model 12 had a slightly better fit (in terms of PPC statistics).

However, looking at the best fit for samples using the ‘past week’ focal instruction on 10-item D-FAW only (Samples 1 and 3), Models 4 and 12 were the best solutions, again with Model 12 showing a slightly better fit (in terms of PPC statistics). Sample 4, which used the ‘today’ focal instruction, was best represented by the Model 6 structure. Hypothesis 2 cannot be supported overall, because although the NA and PA structure (Model 4) showed a good fit with summative ‘past week’ focal instructions, Model 12 was the best fit overall. For Sample 4, the discrete five-factor solution (Model 6) was the best fit.

#### Best-fit model for 10-items extracted from 30-item D-FAW

The best-fit solution for Sample 2 was Model 12; however, the PPC was significant and not all loadings were significant. No model had non-significance for the PPC. The next-best solution was Model 4, but some loadings were non-significant in the opposite direction. As the best-fit model for the ‘past week’ summative focal instruction was also the best fit for Sample 2, Hypothesis 3 is not supported. However, it is noted that Models 4 and 12 are more problematic in this sample. Model 6, which was the best fit overall across all of the samples, also returned one non-significant loading that was in the direction opposite to that hypothesized (–.05).

#### Post hoc analyses

It is possible that there are differing factor structures for within- and between-person portions of multilevel models (Rush and Hofer, 2014^6^). Rush and Hofer’s findings could reflect the influence of response bias at the item level obscuring true factor structures at different levels of analysis. We therefore conducted a series of tests to examine whether within- and between-person factor structures were different using Samples 4, 5 and 6.

Because of the superior fit of models that specified response bias factors, we tested only variants of those models. We conducted the analyses in two steps. In the first step, we analysed within-person variance only and fitted Models 2, 4, 6, 8, 10, 12 and 14. We found Models 6 and 12 to be the best-fitting models. Model 6 had the lowest DIC in Sample 4 and second lowest DIC in Samples 5 and 6. Model 12 had the lowest DIC in Samples 5 and 6, and the second lowest DIC in Sample 4. We retained models 6 and 12 for further consideration. We then ran hybrid models. We ran model 6 or 12 specified for the within-person portion of the model with every combination of Models 2, 4, 6, 8, 12 and 14 in Samples 4, 5 and 6 in the between portion. In Sample 4, the PPC was not significant, indicating that all models had reasonable fit. In Sample 5, the PPC was significant at *p* < .05, and in Sample 6, all models were significant at *p* < .01. In no instance did we find the same model having the lowest or second-lowest DIC across all three samples. Indeed, only one model was ranked lower than 7th on the DIC in all three samples, which was Model 6 for the within-person level with Model 14 at the between-person level (lowest in Sample 4, fourth lowest in Sample 5 and sixth lowest in Sample 6). We label this model 6w/14b. After ranking each model in each Sample by its DIC and summing the ranks across the samples, Model 6w/14b had the lowest combined ranking. Model 6w/14b specifies five first-order factors at the within-level and, at the between-level, five first-order factors loading on two second-order factors corresponding to NA and PA. For Model 6w/14b, all items load in the hypothesized direction in all samples, and all free loadings were significant in Samples 5 and 6. Some 13 out of 14 free loadings were significant in Sample 4.

The results would suggest that, after controlling for response bias factors in the present occupational samples, there is the same first-order factor structure at both within- and between-levels of analysis, but that the second-order factors of NA and PA are more prominent at the between-person level of analysis.

### Study 1 Discussion

Across all of the samples, Model 6 (the five-factor solution, accounting for response bias) was judged to be the best-fit model for assessing AWB using the short-form 10-item D-FAW. This is based on the parsimony and fit indices; Model 6 also follows an established theoretical structure (Daniels, 2000). Figure 3 (available online as supplementary material) illustrates this structure, and provides item loading ranges across the samples. Full details of the item loadings for Model 6 across samples are presented in a table in Appendix 3 (available online as supplementary material). Model 6 was the best-fitting structure for Samples 4 and 5 and the second-best fit for Sample 6. These samples utilized two different focal instructions (daily summative: Sample 4; and momentary: Samples 5 and 6) in within-persons designs. As Model 6 reflects the best-fitting first-order structure of the 30-item version of D-FAW, indications are that when a scale is shortened the underlying first-order factor structure does not change, when the remaining items adequately represent the construct of affect (balanced in terms of valence/hedonic tone and activation). However, when the summative focal instruction, ‘over the past week’ was used, Model 6 was not the best-fitting structure. This suggests that the focal instruction, rather than short scales, is responsible for how we conceptualize affect in ratings.

It also suggests that there is an interaction between scale length and focal instruction. Shortening a scale *and* restricting the recall period (to ‘right now’ or ‘today’) means that discrete emotion is more likely to be captured. However, shortening a scale and using a longer recall period is more likely to capture summed affect or more abstract factors, for example, PA and NA.

The ‘hybrid’ empirical model – Model 12 (included because it was originally tested for the long-form D-FAW) – was also a good overall fit for the data across the six samples. This structure uses four factors: three discrete factors – AC, AP and DP (NA terms) – with a combined fourth factor for PA (incorporating BE and TV). This suggests that people may more memorably recall specific negative emotions when retrospectively rating them, whereas when considering positive affect, they may be more likely to recall a general experience of positive valence/hedonic tone without considering discrete emotions as the rating period lengthens.

#### Extracting items from a long scale

The best-fit model for Sample 2 was Model 12, but not all loadings were significant and the model fit was significant (not desirable). The next-best-fitting model was Model 4, but again loadings were problematic (non-significant or in the opposite direction). This suggests that when items are rated by participants, but then removed from analyses, the structure of the construct remaining can be distorted.

## Study 2

In Study 2, to further examine the construct validity and integrity of using a short-form measure of AWB, the best-fitting structure of the 10-item D-FAW (arranged according to Model 6 in Study 1) was subjected to a convergent construct validity analysis. Although a number of short scales of affect exist, some of these are limited in that they only examine affect as a ‘mood’ construct (level 2, Figure 1), such as POMS-15 (Cranford et al., 2006) and the MDMQ (Wilhelm and Schoebi, 2007). Other measures, such as SPANE, whilst only containing 12 items, include terms that are not representative of discrete emotions, such as ‘bad’, ‘good’, ‘positive’ and ‘negative’ (Diener et al., 2010). Such measures are unsuitable for establishing construct validity with 10-item D-FAW, with its balance of items across the AWB domain, and flexible instruction focus for capturing affect at all levels represented in Figure 1. The 20-item PANAS represents affect in terms of two orthogonal factors of Positive Activated Affect and Negative Activated Affect. PANAS was originally developed by testing six samples across a range of focal instructions capturing momentary, summative and general affect (Watson et al., 1988), and so is appropriate to examine here. It also contains a range of discrete emotion terms organized into higher-order factors allowing for the specific and non-specific measurement of affect (Watson and Clark, 1997).

Despite the prevalent use of PANAS in AWB measurement, concern has been expressed that PANAS does not capture low PA concepts such as fatigue (Diener and Emmons, 1984), and that lower-order specific affect terms are not well understood, in terms of their interrelatedness, especially with regard to positive affect (Watson and Clark, 1997). Daniels (2000) also argues that PANAS has more items representing anxiety than anger in measuring NA.

Daniels’ (2000) second-order factors indicate that AC and AP load onto an NA second-order factor that is roughly equivalent to PANAS NA. However, whereas PANAS NA has more items representing anxiety than anger, D-FAW has an equal number of items representing anger (AP) and anxiety (AC). This suggests that AC will converge more strongly, compared with AP, with PANAS NA. Daniels’ (2000) BE and TV factors load onto a PA second-order factor that is roughly equivalent to PANAS PA. However, as PANAS PA appears to underrepresent fatigue/tiredness at the low end of the scale, it is likely that BE will converge more strongly, compared with TV, with PANAS PA. In light of this, the following hypotheses were formed:

*Hypothesis 4*: D-FAW AC and AP scales will show significant convergent validity with PANAS NA scale, with AC being the strongest predictor, in terms of the relative size of the coefficient and amount of variance captured.
*Hypothesis 5*: D-FAW TV and BE scales will show significant convergent validity with PANAS PA scale, with BE being the strongest predictor, in terms of the relative size of the coefficient and amount of variance captured.

Depression–pleasure is a dimension of AWB, and yet different theoretical positions place it either with positive affect measures (Tellegen, 1985) or with negative affect measures (Russell, 1980). Watson et al. (1988) and Daniels (2000) suggest that DP might be equally suited to either PA or NA dimension. Therefore:

*Hypothesis 6*: D-FAW DP Scale will show significant convergent validity with both PANAS PA and NA, but will have a smaller coefficient size and explain less of the variance than the other predictor variables.

### Method

#### Sample

Forty-six out of 86 UK graduates, working for a large, multinational, IT manufacturing and design organization agreed to participate in a study of well-being at work, and 39 usable response packs were received. Twenty-six participants (67%) were male, the average age was 25.87 years (Range from 22 to 35 years; SD = 3.06), and the average length of time with the company was 2.83 years (Range from 3 months to 6 years; SD = 2.09). This sample is the same sample used for Sample 5 in Study 1 (see Table 1).

#### Materials and procedure

All participants were asked to complete individual article well-being forms three times a day for five consecutive work days. After accounting for five missed instances (random), this gave a total of 580 ratings from 39 participants. Each well-being form contained the D-FAW 10-item measure (see Study 1) and the 20-item PANAS. Ten items used in PANAS represented NA (Distressed, Upset, Guilty, Scared, Hostile, Irritable, Ashamed, Nervous, Jittery, Afraid) and 10 items used in PANAS represented PA (Interested, Excited, Strong, Enthusiastic, Proud, Alert, Inspired, Determined, Attentive, Active). Both measures contained the focal instruction, ‘Indicate to what extent you feel this way *right now, that is, at the present moment*’ (Watson et al., 1988). PANAS PA and NA are scored on 1–5 response rating scales, where 1 = very slightly and 5 = extremely. D-FAW items are scored on 1–6 response rating scales, where 1 = not at all and 6 = very much. The term ‘Active’ is used in both PANAS and the D-FAW 10-item measure, and was the only repeated term.^7^ Variables on the D-FAW were scored in the same direction as the PANAS factors to which they were hypothesized to load most strongly (e.g. a high score on D-FAW AC would correspond to a high score on PANAS NA). DP was scored in the direction of PA.

#### Analysis

The data had a multilevel arrangement, with 580 (max 566 with missing data) well-being reports at level 1 (*i*) and 39 participants at level 2 (*j*). Hierarchical linear modelling (HLM: Kreft and deLeeuw, 2004; Snijders and Bosker, 2004) was applied using MLwiN version 2.36 (Rasbash et al., 2016) in all analyses. All well-being scores were converted to *z*-scores in order to standardize them (as PANAS and D-FAW used different rating scales). All variables were then person-mean-centred in order to limit the impact of potential bias (e.g. self-report) factors on the results (Dimotakis et al., 2011). Having established that a two-level model was a better fit for the data than the null model, the predictor variables were entered, as fixed coefficients (intercepts-only). A random coefficient model (slopes and intercepts) was not tested because of the relatively low sample size at level-2. When level-2 sample sizes are lower than 50, it is recommended that level-2 clustering of effects within the model is avoided to reduce the likelihood of committing type 1 errors, owing to underestimation of variance and standard error (Hox, 1998; McNeish, 2016; McNeish and Stapleton, 2016; Maas and Hox, 2005). In Step 2, DP was removed from the model, in order to assess the impact of removal on the model fit. Outcome variables were for PANAS scales (NA and PA). Predictor variables from D-FAW were AC and AP (Hypothesis 4), and BE and TV (Hypothesis 5). DP was entered as a predictor in both models (Hypothesis 6).

To establish how much of the variance in the model was captured by the predictors in each case, a random intercepts-only model (for Model 1 and then Model 2) was run with fixed coefficients allowed for the predictor variables. In each model, all three predictor variables were entered together after running the null model. The unexplained variance for the model was calculated by summing within- and between-persons unexplained variance (which MLwiN specifies in all multilevel random intercepts equations). On three separate occasions, each of the predictors was then removed from the model, in order to examine the differential impact of each on the total unexplained variance.

### Results

#### Descriptive statistics

Sample 5 in Table 2 (available online as supplementary material) reports the descriptive statistics for the 10-item D-FAW, as used in this study. Table 4 (available online as supplementary material) presents the descriptive statistics for the 20-item PANAS used with the same sample. The 10-item D-FAW is more normally distributed than PANAS – especially compared with PANAS NA, which is beyond acceptable limits for kurtosis (3.87: indicating a very flat distribution) and skewness (1.49: indicating a positive skew). Cronbach’s alpha values (calculated on repeated-measures data using SPSS) for the second-order factors of D-FAW PA and NA are .66 and .80, respectively (based on 4-item scales: negative items reverse-scored). When DP is added to D-FAW PA, the alpha for this scale is .80, and .85 when added to the D-FAW NA scale. Similarly, multilevel alpha (estimated using M-Plus, see Geldhof et al., 2014) revealed that the D-FAW NA and PA scales have higher internal consistency than the discrete two-item scales, and all reach conventional levels of acceptability when the DP items are added.^8^ See Table 5 (available online as supplementary material). This compares with the PANAS alpha values of .92 (10-items) and .81 (10-items) for PA and NA, respectively, calculated using Cronbach’s alpha of within-person variation. Multilevel alphas were estimated at .89 for within-person PA, .85 for between-person PA, .76 for within-person NA and .87 for between-person NA.^9^ We refer the reader to the discussion about alpha as a potentially inappropriate calculation of internal consistency in very short scales in the Introduction.

#### Models

Two models are presented in Table 6 (available online as supplementary material). PANAS NA as an outcome is used in Model 1, and PANAS PA as an outcome is used in Model 2. Model 1 indicates that D-FAW AC (γ = .45, *p* <.01), AP (γ = .17, *p* <.01) and DP (γ = –.14, *p* <.01) scales are significant predictors of NA, improving the Model fit from the null (Δ χ^2^ = 435.74; 3 d.f.; *p* < .001). AC had the largest regression coefficient, over two and a half times the size of the others, consistent with Hypothesis 4. When DP was removed in Step 2, the difference in Model fit between Step 1 and Step 2 was significant (Δ χ^2^ = 12.68; 1 d.f.; *p* < .001). The 2* log likelihood was lower in the Step 1 model, however, suggesting that the best-fitting model for predicting PANAS NA includes AC, AP and DP as parameters. Although statistically significant, DP has the smallest regression coefficient supporting Hypothesis 6.

In Model 2, BE (γ = .43, *p* <.01), TV (γ = .27, *p* <.01) and DP (γ = .17, *p* <.01) from D-FAW are significant predictors of PANAS PA, improving the Model fit from the null (χ^2^ = 622.52; 3 d.f.; *p* < .001). BE had the largest regression coefficient – nearly 60% larger than the next-largest coefficient, consistent with Hypothesis 5. When DP was removed in Step 2, the difference in Model fit between Step 1 and Step 2 was significant (Δ χ^2^ = 27.26; 2 d.f.; *p* < .001). As before, the 2* log likelihood was lower in the Step 1 model, however, suggesting that the best-fitting model for predicting PANAS PA includes BE, TV and DP as parameters. Although statistically significant, DP has the smallest regression coefficient, supporting Hypothesis 6.

Models 1 and 2 were run again to examine the impact of each predictor on the models’ unexplained variance (see Analysis section above). When AC was removed from Model 1, the unexplained variance increased from .68 to .76 (Δ variance = .07). When AP was removed from Model 1, the unexplained variance increased from .68 to .69 (Δ variance = .01). When DP was removed from Model 1, the unexplained variance increased from .68 to .69 (Δ variance = .01). AC predicts most of the variance in PANAS NA, providing support for Hypothesis 4, and consistent with the relative size of the regression coefficients reported in Table 6 (available online as supplementary material). DP and AP account for the smallest proportion of variance, which is not entirely consistent with Hypothesis 6.

In Model 2, the same process was followed to examine the impact of BE, TV and DP on PANAS PA. When BE was removed from Model 2, the unexplained variance increased from .61 to .68 (Δ variance = .07). When TV was removed from Model 2, the unexplained variance increased from .61 to .64 (Δ variance = .03). When DP was removed from Model 2, the unexplained variance increased from .61 to .62 (Δ variance = .01). BE predicts most of the variance in PANAS PA, providing support for Hypothesis 5, and consistent with the relative size of the regression coefficients reported in Table 6 (available online as supplementary material). DP accounts for the smallest proportion of variance, consistent with Hypothesis 6.

In both models, DP is a significant but lesser predictor of PANAS NA and PA (supporting Hypothesis 6) compared with the other scales, although this is only marginally the case in respect of AP in Model 1. The model fit (Table 6) for predicting PANAS NA and PA is stronger in both cases when DP is included (Step 1 models). The change in χ^2^ from the null model to the Step 1 model is greater in Model 2, suggesting that DP may offer a slightly better solution when used as a predictor of PANAS PA than PANAS NA.

### Study 2 Discussion

Overall, the results of Study 2 demonstrate good convergent construct validity for D-FAW with an established measure of affect (PANAS), when D-FAW is presented in its 10-item short form. D-FAW AC, AP and DP scales are significantly associated with PANAS NA. In particular, the predictive strength of AC in the model reveals that Anxiety–Comfort items are dominant in PANAS, providing support to Hypothesis 4. D-FAW TV, BE and DP scales are also significantly associated with PANAS PA, with BE more predictive than TV, supporting Hypothesis 5. This confirms that fatigue items are less well represented in PANAS (Watson and Clark, 1997), despite being a feature of low activated affect (Larsen and Diener, 1992). In both models, DP was a significant predictor but to a lesser extent than the other two scales, supporting Hypothesis 6. Although both models showed a significant reduction in fit when DP was removed in Step 2, DP provided a greater change in fit from the null model at Step 1, in the PA model compared with the NA model. It also more greatly enhanced the alpha coefficient of the D-FAW PA scale to an acceptable level (Nunnally, 1978) for within- and between-person alphas (see Table 5, available online as supplementary material). As such, given a choice, DP is likely to be best included in models that predict PA, rather than NA.

This study demonstrates that we can confidently apply the 10-item short-form D-FAW as a comprehensive measure of AWB in an applied context. Further, D-FAW appears to tap into constructs that are less well represented in PANAS (such as Anger and Fatigue). Finally, the more normal distribution of scores for 10-item D-FAW, compared with 20-item PANAS, indicates that response bias is less of a problem with D-FAW, potentially because of the balance of negative and positive items for each dimension and construct (Daniels, 2000).

## Overall discussion

Psychological well-being is increasingly moving towards the status of being an essential component in understanding the myriad of work experiences studied within applied, organizational research. As a key component of psychological well-being, AWB is more prominent within the research literature than ever before. Measures of AWB therefore need to be valid and reliable in order that researchers and policy-makers can trust the results of the studies in which they are used.

In the present article, we focused on the need to use short-form standalone scales of AWB in organizational studies, given the increasing employment of repeated-measures and/or multi-scale designs. In shortening AWB scales, we argued that these still need to be comprehensive, flexible in their focal instructions, and able to maintain their psychometric integrity. Researchers frequently recognize the need to shorten scales in organization studies but do not always consider how removing items from long-form scales, or simply constructing new short-form measures, will impact on the underlying factor structure or validity of the scales.

### Shortening scales and the impact on validity and psychometric integrity

Across two studies, utilizing the short-form 10-item version of the D-FAW (Daniels, 2000), we demonstrate that affect can still be represented comprehensively as comprising five key facets of affect, balanced in terms of the two key well-being components: hedonic tone/valence and activation. The overall best-fitting model for the short-form D-FAW measure of momentary AWB used in repeated-measures designs indicates a single-level structure of discrete emotion factors (see Figure 3, available online as supplementary material). This fits with the structure of affect reported by Weiss and Cropanzano (1996), Brief and Weiss (2002) and Frijda (1993). A mood-based grouping of PA and NA is normally considered to be structurally representative at the summative level, but we found that the five-factor structure also best explained how affect is organized when rating on a daily (summative) basis. Previous studies have utilized PA/NA or other mood-based summaries when looking at how people rate their affect on a daily basis (Beal et al., 2013; Beal and Ghandour, 2011; Louro et al., 2007). This research indicates that it may be more appropriate to use discrete factors when examining daily affect in future. Post hoc analysis revealed that Model 6 is a better fit for within-person differences, with Model 14 the best fit for explaining aggregated between-person differences in momentary measures.

Because Model 12 and then Model 4 (NA and PA) are the best-fit models for summative ‘past week’ focal instructions on 10-item administrations only, this indicates that at some point between rating over the past day and rating over the past week, people move from considering affect as individual, discrete emotion factors and begin to sum their feelings about work. It will be interesting for researchers to now explore at what point people move from summarizing their emotions in discrete terms to general terms – is it on timeframes longer than one day, two days, seven days? The summing of emotions over longer periods of time could be a function of memory recall (Reis and Gable, 2000), as we may focus on a general memory of our hedonic tone or activation levels when remembering mood and events across extended timeframes, and specificity is lost (Ilies et al., 2015; Xanthopoulou et al., 2012). It will be interesting to further explore whether specificity in the recall of affect is biased towards negative events (Miner et al., 2005; Taylor, 1991). Although Model 12 does not reflect an established theoretical structure of affect, it suggests the possibility that how we recall and summarize emotions may transcend from discrete focus at momentary and short-term levels (‘now’ and ‘today’) to a discrete focus on negative activated affect but summed positive activated affect (‘past week’), and then to a mood-based PA and NA solution. Further research would elucidate this, although we emphasize that currently Model 12 has only empirical, rather than conceptual, support.

### Item context and the structural representation of affect

Using a summative focal instruction that extracted ten items from the 30-item D-FAW administration revealed a factor structure that had problems in terms of fit and how some of the items loaded. Researchers frequently extract items from long-form measures, to suit the purpose of their study, rather than using standalone short-form measures (e.g. Harmon-Jones, 2003; Ouweneel et al., 2012). Our results indicate, however, that the context of other terms used when making an assessment of affect may matter significantly. When contextualized with many other emotion terms, mood may become summarized. However, when raters focus on fewer distinctive terms in a standalone measure, affect may be seen as more specific and distinct. Without other terms to anchor the meaning we apply to affect, the underlying conceptualization (made by participants as they navigate a scale) might change. This is then reflected in the factor structure when tested. This was especially salient when looking at the factor structure of D-FAW used in Sample 2. By removing 10-items out of the context in which they were originally interpreted (the 20 discarded items), the underlying factor structure was very difficult to fit, and loadings were either non-significant or worked in the opposite manner to that expected. This reflects findings by Hofmans et al. (2008). We suggest that researchers take heed from these results, and ensure that the factor structure of short-form standalone scales is checked before the scales are used in an applied setting.

### Limitations

In Study 1, we used samples from different organizational research settings. Therefore, there was contextual variation across samples, precluding strict equivalence. Furthermore, because all of our samples used D-FAW in an occupational context, it may be the case that conclusions made in this article cannot be extended to other domains where AWB is of interest, such as in educational or health settings. To extend our findings to the issues affecting the generic measurement of AWB, D-FAW would need to be validated in alternative contexts.

Further, the samples completing the short form of D-FAW in Study 1 were not matched with the composition of the original samples used in the Daniels’ (2000) long-form validation samples. Therefore, we cannot be sure that the factor structure of long-form D-FAW is or is not consistent with short-form D-FAW as a result of the different samples used. Nevertheless, by using a range of samples in this article, with ratings gathered from working adults across a variety of industry sectors, with a range of genders and ages, we are reassured that the generalizability of the best-fitting factor structures uncovered here would be replicated with other working adults completing the short-form D-FAW.

Finally, in our multilevel data, the level-2 *N* was not always as high as we would like. Whilst low *N* is relatively common in repeated-measures studies conducted in applied settings (see Conway and Briner, 2002: *N* = 45; Elfering et al., 2005: *N* = 23; Miner et al., 2005: *N* = 41), it is not ideal. Our lowest *N* was 36 (Sample 4). Our use of B-CFA, as opposed to CFA, is advantageous in this context, because B-CFA is able to offer more stability when conducting multilevel modelling with small level-2 sample sizes (Muthén, 2010).

## Conclusion and contribution

Our research indicates that shortening a well-being scale for use in measuring AWB will not necessarily compromise psychometric integrity and the comprehensive coverage of affect terms if (a) scales are balanced in terms of hedonic tone/valence and activation; and (b) affect is measured using discrete terms at the momentary level and on a daily timeframe, with mood-based summaries of PA and NA (either as a single factor or as two factors) being more relevant at the summative level focusing on ‘the past week’ or longer.

Our research makes a key contribution in demonstrating that the focal instruction chosen to measure AWB impacts on the underlying factor structure of affect in the short form; this may have been overlooked in previous studies because of the limitations of traditional analysis methods. Using B-CFA, we have shown that five factors, plus response bias factors, fit best when using momentary and summative ‘today’ focal instructions on the 10-item D-FAW. A reductive two- to four-factor structure fits best with a summative ‘past week’ focal instruction in a short-form measure. More research is needed to understand at what point affect moves from being conceived of as distinct, to a summary of mood (whereby negative activated emotions are more clearly observed). Further, we suggest that more work is needed to understand how items contextualize each other in rating AWB. When scales are shortened and items discarded, we have shown that this can impact on underlying factor structures and potentially the meaning attributed to the surviving terms.

Finally, we suggest that these findings are of special relevance to researchers wishing to measure AWB in applied organizational settings. Our understanding of how AWB is related to work events and experiences – (i) in an episodic way, (ii) when using multiple scales and (iii) for cross-level analyses – can be best progressed if the scales used to measure AWB in such contexts are psychometrically robust, flexible in their focal instructions, and conceptually comprehensive.

## Supplemental Material

HUM751034_Supplementary_Material – Supplemental material for Measuring affective well-being at work using short-form scales: Implications for affective structures and participant instructions Click here for additional data file. Supplemental material, HUM751034_Supplementary_Material for Measuring affective well-being at work using short-form scales: Implications for affective structures and participant instructions by Emma Russell and Kevin Daniels in Human Relations

## Appendix

**Appendix 1:** The standalone short-form 10-item Daniels five-factor measure of affective well-being (D-FAW). In the section below, please indicate how you feel *right now, that is, at the present moment****. Please circle the most appropriate number on the six-point scale, where 1 = not at all to 6 = very much.

Happy	1	2	3	4	5	6
At ease	1	2	3	4	5	6
Anxious	1	2	3	4	5	6
Annoyed	1	2	3	4	5	6
Motivated	1	2	3	4	5	6
Calm	1	2	3	4	5	6
Tired	1	2	3	4	5	6
Bored	1	2	3	4	5	6
Gloomy	1	2	3	4	5	6
Active	1	2	3	4	5	6

* This focal instruction can be amended according to timeframe and context.

## Notes

1 A focal instruction is the rating instruction to the participant, which serves to focus attention to a particular context (e.g. ‘rate your experience in relation to *this job* role’). Time-bound focal instructions direct participants’ attention to the period within which they are expected to recall the construct/term in question (e.g. ‘rate your well-being *today* on the following terms’).

2 Although it is unclear as to whether mood, as an aggregate of level 3 emotions, is conceptually equal to mood when measured summatively at level 2 (Beal and Ghandour, 2011).

3 Short scales (unlike *short-form* scales) do not have a long-form equivalent. Short-form scales must be administered in a standalone and standardized format (i.e. a long-form scale should not be administered and then items extracted to represent the short scale post hoc).

4 Except in Sample 3, where ‘Angry’ was used instead of ‘Annoyed’.

5 DP is scored in the direction of PA, that is, Happy (+) and Gloomy (–) unless being used to load onto the NA factor. At such times, the reverse scores are applied.

6 We thank an anonymous reviewer for this observation.

7 The duplication of the term ‘Active’ does not mean that one of the terms becomes redundant because each is positioned and contextualized with the other terms used for each respective measure. We correlated (using repeated measures) Active D-FAW and Active PANAS terms with each other in this sample. The correlation was at a level of .66, suggesting that the two terms are being rated differently by the same person on the same occasion, likely owing to a combination of different scoring bands and context. We also ran a split-half reliability with Spearman-Brown correction on Active D-FAW with PANAS D-FAW, which gave a reliability coefficient of .8. To drop one of the Active terms would be problematic: which should be dropped, and how does this impact either D-FAW or PANAS, given that the context will change if a term is omitted from the original completion format? We therefore retain both items in this study.

8 Although Geldhof et al. (2014) recommend using multilevel composite reliability, we encountered identification problems with Sample 5 in estimating multilevel composite reliabilities. However, Geldhof et al. (2014) do indicate that estimates of multilevel composite reliability and multilevel alpha are relatively unbiased in most cases. Because we did not encounter as many identification problems with multilevel alpha, we report these coefficients.

9 These models were not identified.
