# Supplementary material for: Measuring affective well-being at work using short-form scales: Implications for affective structures and participant instructions
Source: Hum Relat. 2018 Apr 13;71(11):1478–507. doi: 10.1177/0018726717751034 (PMC6146316; doi:10.1177/0018726717751034)
Supplement: HUM751034_Supplementary_Material – Supplemental material for Measuring affective well-being at work using short-form scales: Implications for affective structures and participant instructions [file HUM751034_Supplementary_Material.pdf]

Measuring affective well-being at work using short-form scales: Implications for affective structures and participant instructions

Emma Russell and Kevin Daniels

Table 2: Means, standard deviations, skew and kurtosis values for 10-item Daniels Five-factor measure of Affective Well-being (D-FAW)

Well-being scale	Range	Mean	Standard deviation	Skew	Kurtosis
Sample 1 (SE: 0.15 Skew; 0.31 Kurtosis)					
AC (N=253)	5.00	3.81	1.23	–0.57	–.82
AP (N=253)	5.00	3.82	1.05	–0.14	–.39
BE (N=254)	5.00	4.30	1.29	–0.59	–.59
TV (N=251)	5.00	3.70	1.06	–0.02	–.27
DP (N=252)	5.00	4.22	1.10	–0.66	.08
Sample 2 (SE: 0.06 Skew; 0.12 Kurtosis)					
AC (N=1794)	5.00	3.69	1.07	–0.26	–0.34
AP (N=1794)	5.00	3.70	1.03	–0.18	–0.44
BE (N=1794)	5.00	4.04	1.06	–0.48	–0.09
TV (N=1794)	5.00	3.64	1.02	–0.20	–0.44
DP (N=1794)	5.00	3.94	1.07	–0.33	–0.35
Sample 3 (SE: 0.13 Skew; 0.26 Kurtosis)					
AC (N=340)	4.50	4.04	0.95	–0.30	–0.39
AP (N=340)	4.50	4.24	0.87	–0.38	–0.13
BE (N=340)	4.00	4.45	0.88	–0.51	–0.19
TV (N=340)	4.50	3.96	0.90	–0.39	–0.36
DP (N=340)	4.50	4.30	0.96	–0.54	–0.09
Sample 4 (SE: 0.15 Skew; 0.29 Kurtosis)					
AC (n=284/N=36)	5.00	3.99	1.19/0.92	–0.29	–0.49
AP (n=284/ N=36)	5.00	4.39	1.15/0.91	–0.81	0.26
BE (n=284/ N=36)	5.00	4.80	0.80/0.56	–1.11	2.76
TV (n=284/ N=36)	5.00	4.01	1.13/0.86	–0.38	–0.30
DP (n=284/ N=36)	5.00	4.70	1.05/0.76	–1.16	1.45
Sample 5 (SE: 0.10 Skew; 0.20 Kurtosis)					
AC (n=570/N=39)	5.00	4.36	1.09/0.84	0.68	–0.26
AP (n=569/ N=39)	4.50	4.44	0.97/0.73	0.75	0.11
BE (n=570/ N=39)	5.00	4.32	0.97/0.74	–0.44	–0.32
TV (n=571/ N=39)	5.00	3.79	1.06/0.80	–0.06	–0.42
DP (n=570/ N=39)	5.00	4.74	0.98/0.72	–1.04	0.67
Sample 6 (SE: 0.08 Skew; 0.16 Kurtosis)					
AC (n=927/N=98)	5.00	4.48	1.02/0.57	–0.55	–0.03
AP (n=923/ N=98)	4.50	4.71	0.95/0.59	–0.78	0.22
BE (n=921/ N=98)	5.00	4.40	0.96/0.47	–0.51	–0.07
TV (n=925/ N=98)	4.50	3.74	1.01/0.49	–0.19	–0.26

DP (n=922/ N=98)	5.00	4.88	0.90/0.41	–0.93	0.55

Notes: AC = Anxiety–Comfort; AP- Angry–Placid; BE = Bored–Enthusiastic; TV = Tiredness–Vigor; DP = Displeasure–Pleasure; SE = Standard Error; n/N given is the minimum overall; all scales are scored in the positive direction so that a high score indicates positive well-being; data from multi-level samples (4, 5 and 6) is provided at level-1; For samples 4–6 standard deviations (SDs) are reported for level-1 and then level-2 data, with level-2 SDs calculated by taking the SD for each individual's set of level-1data and then averaging these across N cases at level-2.

Table 3: Bayesian fit and convergent statistics for different models

Model number	Fit statistics	Sample 1	Sample 2	Sample 3	Sample 4	Sample 5	Sample 6
1. Single factor	PSR	= 1.01	= 1.02	= 1.01	= 1.02	= 1.01	= 1.02
Overall well-being: OWB	PPC	= p < .01	= p < .01	= p < .01	= p < .01	= p < .01	= p < .01
	DIC	= 7912.46	= 53210.77	= 9127.95	= 7300.67	= 14876.29	= 19182.73
	Loadings	+	+	+	+	? (17/18)	+
2. Single factor with response bias factors	PSR	1.04	= 1.02	**This model would not converge even after increasing convergence criterion from .01 to .05**	= 1.02	**This model would not converge even after increasing convergence criterion from .01 to .05**	**This model would not converge even after increasing convergence criterion from .01 to .05**
	PPC	= p < .01	= p < .01		= ns (p >.05)		
	DIC	= 7746.91	= 51474.95		= 7173.86		
	Loadings	-	--		? (17/18)		
3. Two factor	PSR	= 1.01	= 1.02	= 1.01	= 1.02	= 1.02	= 1.02
NA = AC, AP and DP	PPC	= p < .01	= p < .01	= p < .01	= p < .01	= p < .01	= p < .01
PA = TV and BE and DP	DIC	= 7820.04	= 52827.49	= 9058.48	= 7272.7	= 14560.64	= 19016.07
	Loadings	+	+	+	? (17/20)	? (19/20)	+
4. Two factor with response bias factors	PSR	= 1.02	= 1.02	= 1.02	= 1.02	= 1.02	= 1.02
	PPC	= p > .10	= p < .01	= ns (p > .20)	= ns (p > .13)	= p < .05	= p < .01
	DIC	= 7722.45	= 51396.77	= 8831.59	= 7162.65	= 14397.32	= 18893.87
	Loadings	? (11/12)	-	? 6/8	+	? (19/20)	? (18/20)
5. Discrete first-order factors	PSR	= 1.01	= 1.02	= 1.02	= 1.02	= 1.02	= 1.02
AC, AP, BE, TV and DP	PPC	= p < .01	= p < .01	= p < .01	= p < .01	= p < .01	= p < .01
	DIC	= 7814.21	= 52524.69	= 9046.34	= 7260.04	= 14523.66	= 19016.07
	Loadings	+	+	+	+	? (9/10)	+
6. Discrete first-order factors with response bias factors	PSR	= 1.02	= 1.02	= 1.02	= 1.02	= 1.02	= 1.02
	PPC	= p > .15	= p < .01	= p < .05	= ns (p > .15)	= p < .05	= p < .01
	DIC	= 7726.82	= 51416.18	= 8845.68	= 7145.43	= 14394.31	= 18875.76
	Loadings	+	+	=	+	+	+
7. Discrete first-order factors loading onto one second order factor	PSR	= 1.02	(C) = 1.08	= 1.02	= 1.02	= 1.02	= 1.02
	PPC	= p < .01	= p < .01	= p < .01	= p < .01	= p < .01	= p < .01
	DIC	= 7863.83	= 52934.6	= 9103.26	= 7291.54	= 14799.81	= 19150.24
AC, AP, BE, TV and DP; OWB	Loadings	+	+	+	+	? (19/20)	+
8.. Discrete first-order factors loading onto one second order factor with response bias factors	PSR	= 1.02	= 1.02	= 1.02	= 1.02	= 1.02	= 1.02
	PPC	= p < .01	= p < .01	= ns (p > .08)	= ns (p > .11)	= p < .01	= p < .01
	DIC	= 7748.71	= 51403.91	= 8842.54	= 7158.08	= 14405.03	= 18896.25
	Loadings	?	--	+	? (8/10)	-	--

9. <u>Discrete first-order factors for</u>	PSR	= 1.01	= 1.02	= 1.01	= 1.02	= 1.02	= 1.02
<u>PA items</u> <u>and single NA factor</u>	PPC	= p < .01	= p < .01	= p < .01	= p < .01	= p < .01	= p < .01
BE, TV and DP and NA	DIC	= 7818.11	= 52563.47	= 9045.25	= 7257.25	= 14565.82	= 19025.05
	Loadings	+	+	+	+	? (11/12)	+
<u>10. Discrete first-order factors for</u>	PSR	= 1.02	**This model would**	**This model would**	= 1.02	= 1.02	= 1.02
<u>PA items and single NA factor</u>	PPC	= p < .05	**not converge even**	**not converge even**	= ns (p > .18)	= p < .05	= p < .01
<u>with response bias factors</u>	DIC	= 7730.38	**after increasing**	**after increasing**	= 7147.93	= 14398.58	= 1888.83
	Loadings	?	**convergence**	**convergence**	? (11/12)	? (10/12)	? (11/12)
			**criterion from .01 to**	**criterion from .01 to**			
			**.05**	**.05**			
<u>11. Discrete first-order factors for</u>	PSR	= 1.02	= 1.02	= 1.01	= 1.02	= 1.02	= 1.02
<u>NA items and single PA factor</u>	PPC	= p < .01	= p < .01	= p < .01	= p < .01	= p < .01	= p < .01
AC, AP and DP and PA	DIC	= 7813.26	= 526189.13	= 9063.16	= 7256.16	= 14530.06	= 19012.81
	Loadings	+	+	+	? (11/12)	+	+
<u>12. Discrete first-order factors for</u>	PSR	= 1.02	= 1.02	= 1.02	= 1.02	= 1.02	= 1.02
<u>NA items and single PA factor</u>	PPC	= ns (p > .48)	= p < .01	= ns (p > .20)	= ns (p > .16)	= p < .05	= p < .01
<u>with response bias factors</u>	DIC	= 7713.03	= 51366.50	= 8835.52	= 7152.49	= 14394.78	= 18870.16
	Loadings	? 5/6	? (5/6)	? (4/6)	? (11/12)	? (11/12)	?(11/12)
<u>13. Discrete first order factors</u>	PSR	= 1.02	= 1.02	= 1.02	= 1.02	= 1.02	= 1.02
<u>loading onto two second order</u>	PPC	= p < .01	= p < .01	= p < .01	= p < .01	= p < .01	= p < .01
<u>factors</u>	DIC	= 7817.48	= 52647.37	= 9063.79	= 7265.95	= 14560.95	= 19026.07
AC, AP, BE, TV and DP	Loadings	+	+	+	? (11/12)	+	+
PA (with DP) and NA (with DP)							
14. <u>Discrete first order factors</u>	PSR	= 1.02	= 1.02	= 1.02	= 1.02	= 1.02	= 1.02
<u>loading onto two second order</u>	PPC	= ns (p > .05)	= p < .01	= p < .01	= ns (p > .20)	= p < .05	= p < .01
<u>factors with response bias factors</u>	DIC	= 7731.51	= 51407.42	= 8850.50	= 7146.56	= 14399.64	= 18902.02
	Loadings		-	? (8/9)	? (17/18)	? (16/18)	-

Notes: : AC = Anxiety–Comfort; AP- Angry–Placid; BE = Bored–Enthusiastic; TV = Tiredness–Vigor; DP = Displeasure–Pleasure; NA = negative activated affect; PA = positive activated affect; PSR = potential scale reduction; PPC = posterior predictive checking; DIC = deviance information criteria. "C" = (next to PSR) indicates convergence criteria increased to get model to run to conclusion; "+" = All substantive loadings (within- and between- if appropriate) in hypothesized direction and p < .05; "?" (n) = All substantive loadings (within- and between- if appropriate) in hypothesized direction and most p < .05 – numbers in brackets is number of sig loadings/number of potential substantive significant loadings; "-" = Some loadings non-significant in opposite direction; "--" = No loadings significant or some loadings significant in opposite direction. Two response bias factors were fitted– one for positively valenced items and one for negatively valenced items

Table 4: Descriptive statistics for 20-item Positive and Negative Affect Schedule (PANAS)

Scale	Range	Mean	Standard deviation (level 1/level 2)	Skew	Kurtosis
PANAS PA (n=574/ N=39)	5.00	2.94	.83/.74	–.31	.29
PANAS NA (n=575/ N=39)	3.30	1.29	.42/.69	1.49	3.87

Notes: NA = negative activated affect; PA = positive activated affect; Level-2 standard deviations (SDs) are calculated by taking the SD for each individual's set of level-1data and then averaging these across N=39 cases at level-2.

Table 5: Within- and between-person multi-level alpha reliabilities for Daniels Five-factor measure of Affective Well-being (D-FAW)

Scale	Within-person alpha	Between-person alpha	Alpha, estimated through SPSS (within-person)
AC	.55	.46	.67
AP	.59	.78	.55
DP	.59	.76	.66
BE	.49	.43	.59
TV	.50	.24	.31
NA (AC+AP)	.74	.81	.80
PA (BE+TV)	.67	.57	.66
NA + DP	.79*	.87*	.85
PA + DP	.75*	.80*	.80

Notes: * Model not identified; AC = Anxiety–Comfort; AP- Angry–Placid; BE = Bored–Enthusiastic; TV = Tiredness–Vigor; DP = Displeasure–Pleasure; NA = negative activated affect; PA = positive activated affect; SPSS = statistical package for the social sciences.

Table 6: Predicting Positive and Negative Affect Schedule (PANAS) factors of affective well-being (AWB) with factors of 10-item Daniels Five-factor measure of Affective Well-being (D-FAW)

Model 1: Predicting PANAS NA

**Variable**	**Null (2-level)**	**Step 1**	**Step 2**
Intercept	.001 (.099)	.044 (.102)	.043 (.102)
Fixed effects			
AC		.454 (.038)**	.491 (.037)**
AP		.172 (.041)**	.225 (.038)**
DP		-.141 (.039)**	*removed*
Level one variance (within-person)	.670 (.041)**	.301 (.019)**	.309 (.019)**
Level two variance (between-person)	.335 (.087)**	.382 (.092)**	.381 (.092)**
2* log likelihood	1460.538 (N=566)	1024.804 (N=556)	1037.476 (N=556)
Chi-squared difference in model fit		435.74 (3df)**	12.68 (1df)**
		From null	From Step 1

Model 2: Predicting PANAS PA

**Variable**	**Null (2-level)**	**Step 1**	**Step 2**
Intercept	.002 (.104)	.035 (.105)	.034 (.105)
Fixed effects			
BE		.431 (.032)**	.502 (.030)**
TV		.266 (.030)**	.301 (.030)**
DP		.167 (.032)**	*removed*
Level one variance (within-person)	.636 (.039)**	.200 (.012)**	.211 (.013)**
Level two variance (between-person)	.375 (.096)**	.411 (.097)**	.410 (.097)**
2* log likelihood	1437.056 (N=566)	814.532 (N=556)	841.793 (N=556)
Chi-squared difference in model fit		622.524 (3df)**	27.261 (1df)**
		From Null	From Step 1

Notes: DP = Daniels' measure of Depression–Pleasure (happy, gloomy: reversed); AC = Daniels' measure of Anxiety–Comfort (anxious, at ease: reversed); AP = Daniels' measure of Angry–Placid (annoyed, calm: reversed). DP = BE = Daniels' measure of Bored-Enthusiastic (motivated, bored: reversed); TV = Daniels' measure of Tired–Vigour (active, tired: reversed). NA = negative activated affect; PA = positive activated affect; Standard errors are shown in brackets. * = p< .05; ** = p< .01

Figures 2a–g: Factor structures tested for in 10-item Daniels Five-factor measure of Affective Well-being (D-FAW) (and the models represented)

Figure 2a: The first order single factor structure of Daniels Five-factor measure of Affective Well-being (D-FAW) (Models 1* and 2)**

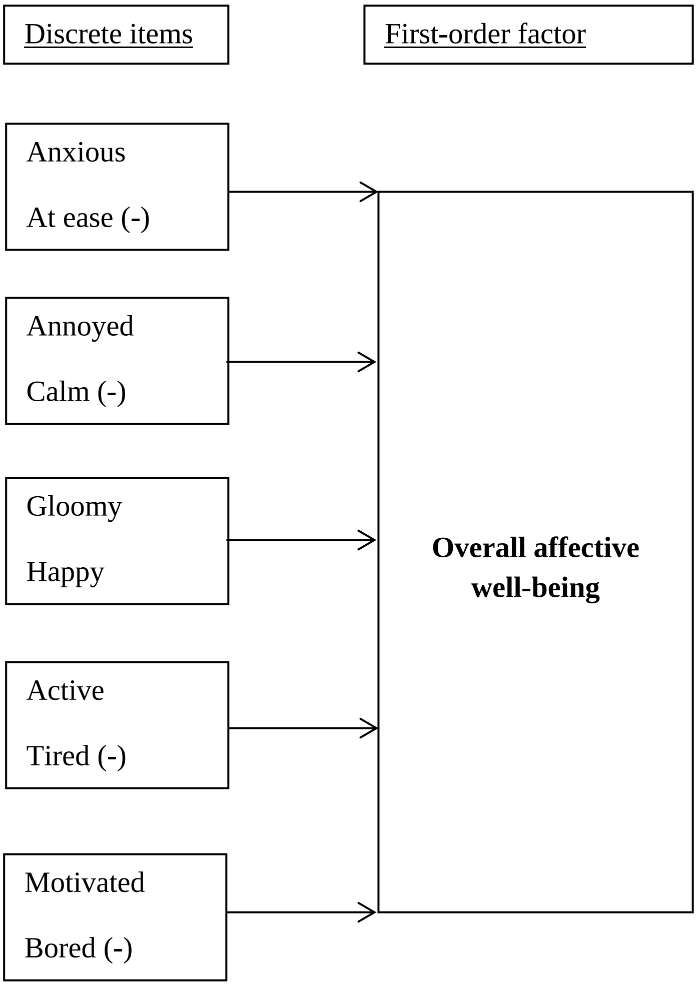

Notes: *without response bias factors; **with response bias factors

Figure 2b: The first order two-factor structure of Daniels Five-factor measure of Affective Well-being (D-FAW) (Models 3* and 4)**

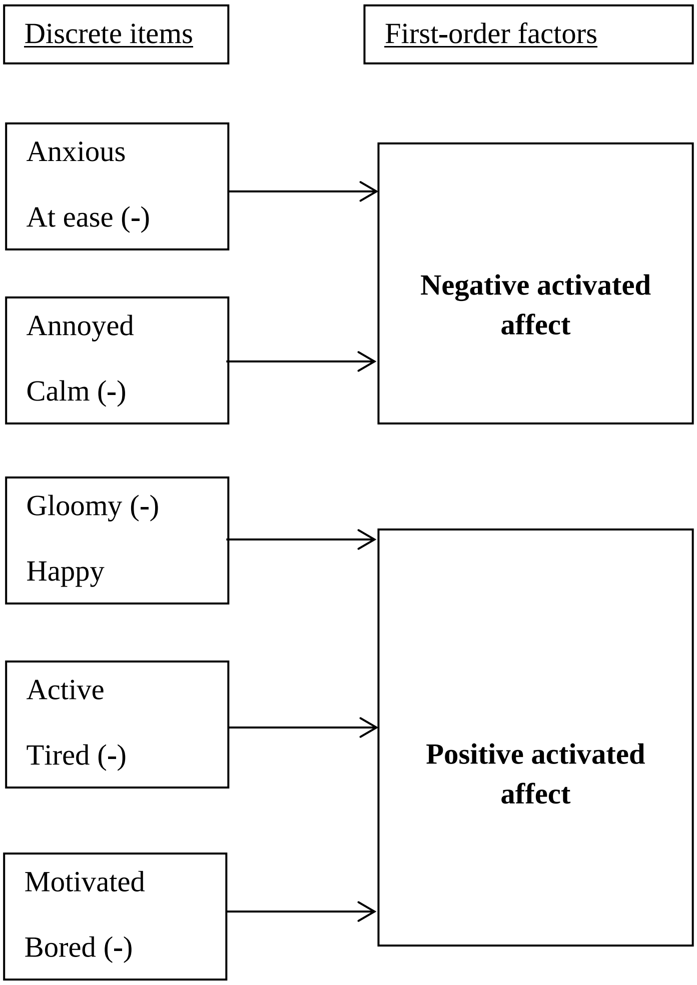

Notes: *without response bias factors; **with response bias factors

Figure 2c: The first order five-factor structure of Daniels Five-factor measure of Affective Well-being (D-FAW) (Models 5* and 6)**

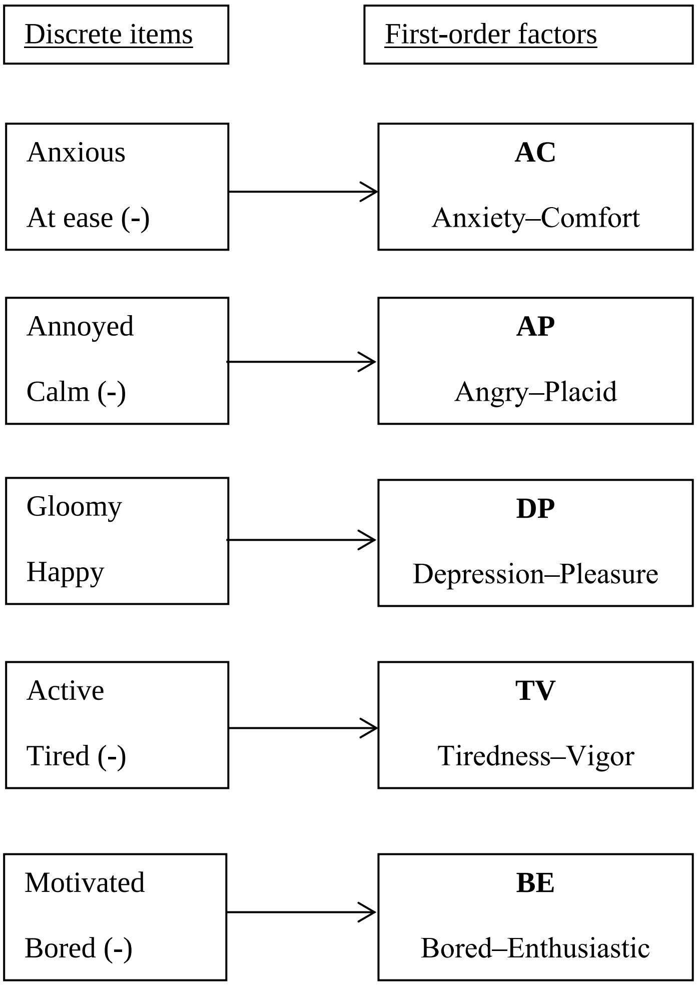

Notes: *without response bias factors; **with response bias factors

Figure 2d: The five-factor first order and single second order factor structure of Daniels Five-factor measure of Affective Well-being (D-FAW) (Models 7* and 8)**

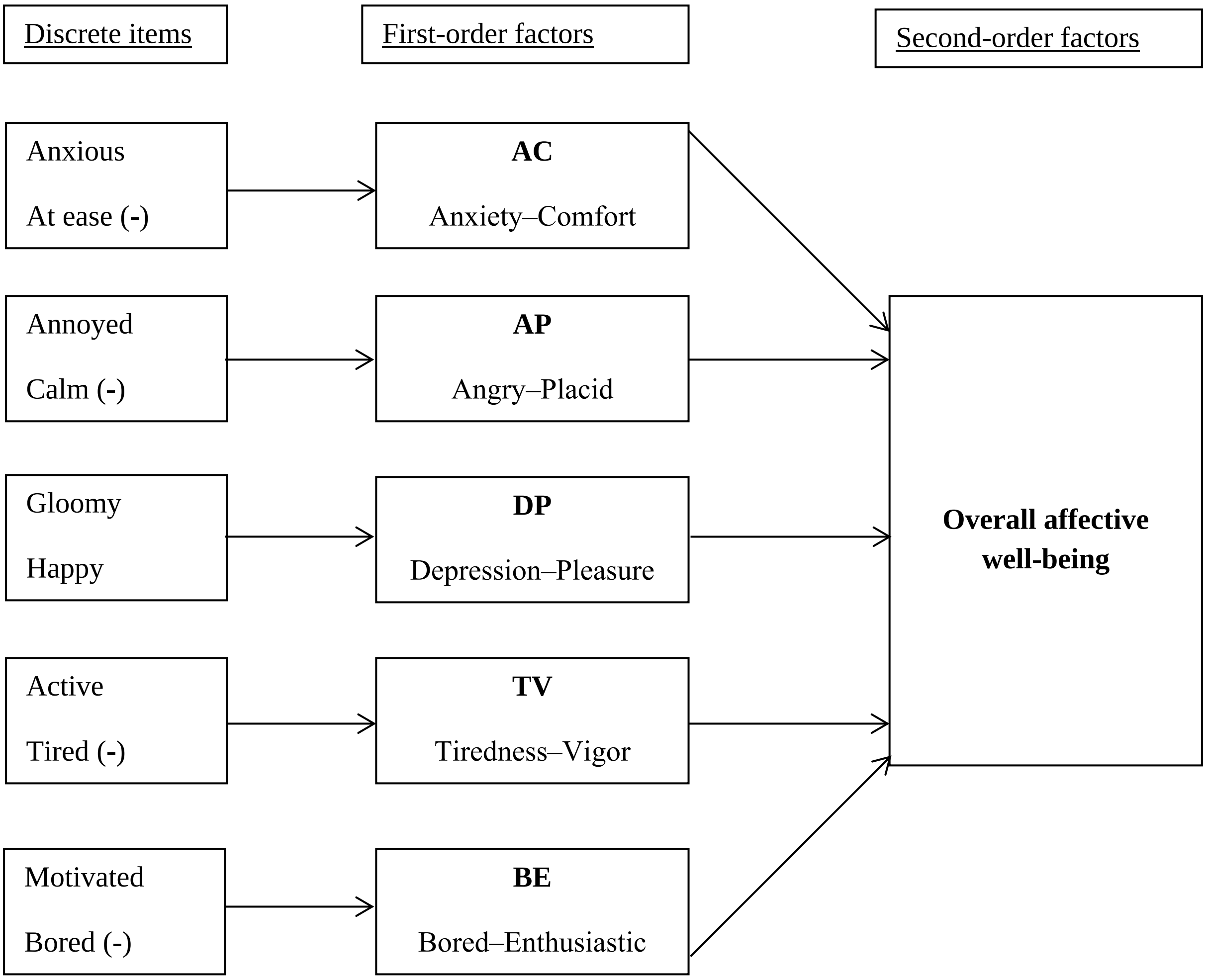

Notes: *without response bias factors; **with response bias factors

Figure 2e: The first order PA-related factors and single NA factor structure of Daniels Five-factor measure of Affective Well-being (D-FAW) (Models 9* and 10)**

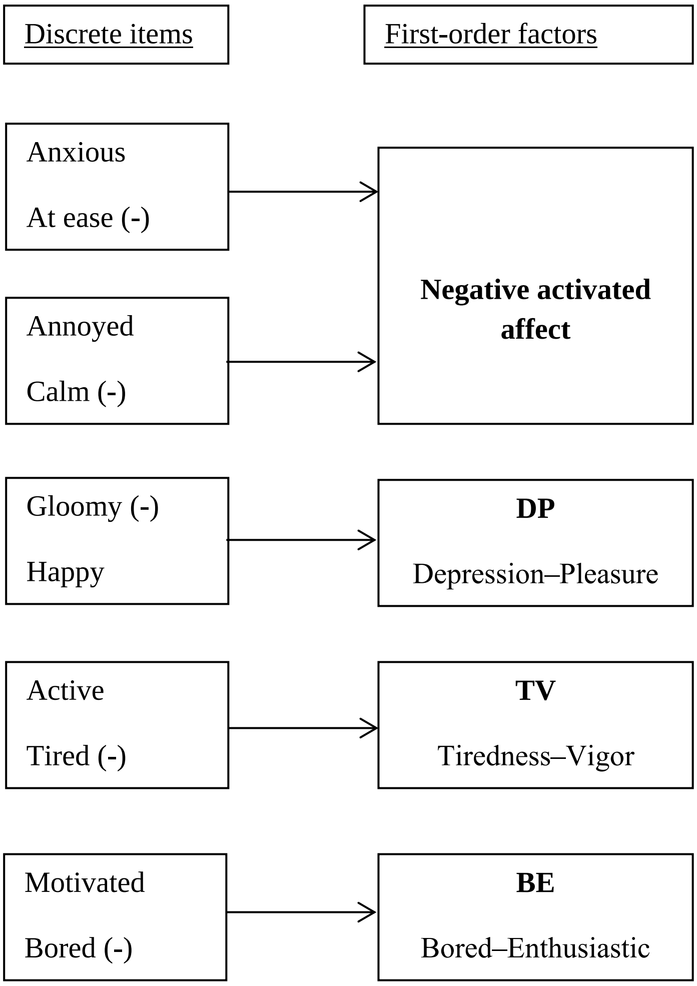

Notes: *without response bias factors; **with response bias factors; NA = negative activated affect; PA = positive activated affect.

Figure 2f: The first order NA-related factors and single PA factor structure of Daniels Five-factor measure of Affective Well-being (D-FAW) (Models 11* and 12)**

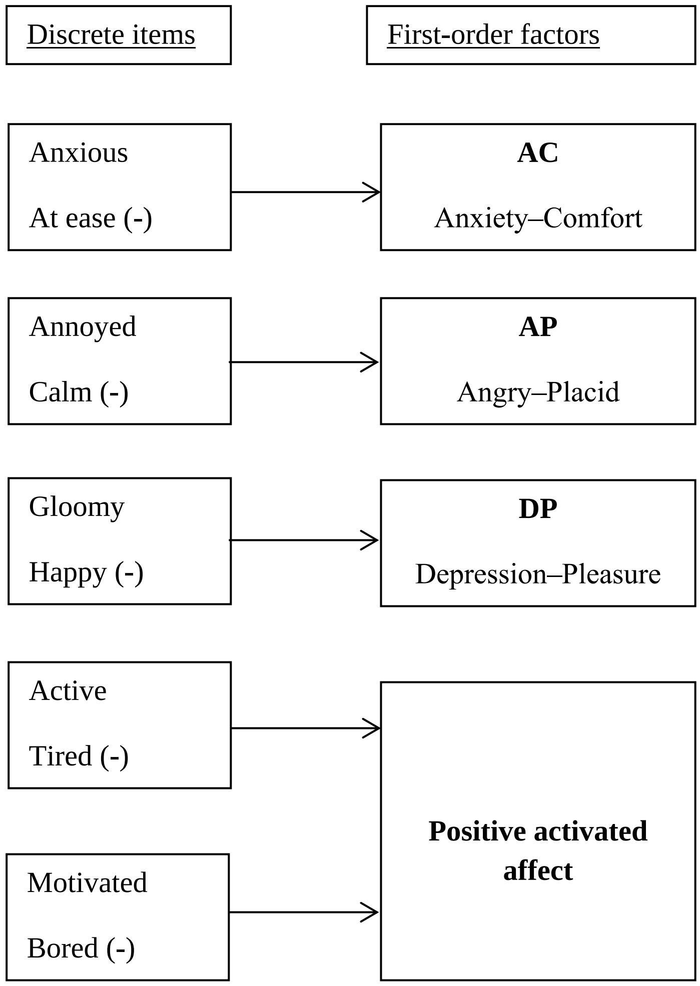

Notes: *without response bias factors; **with response bias factors; NA = negative activated affect; PA = positive activated affect.

Figure 2g: The Daniels Five-factor measure of Affective Well-being (D-FAW) long-form factor structure applied to the 10-item short-form (Models 13* and 14)**

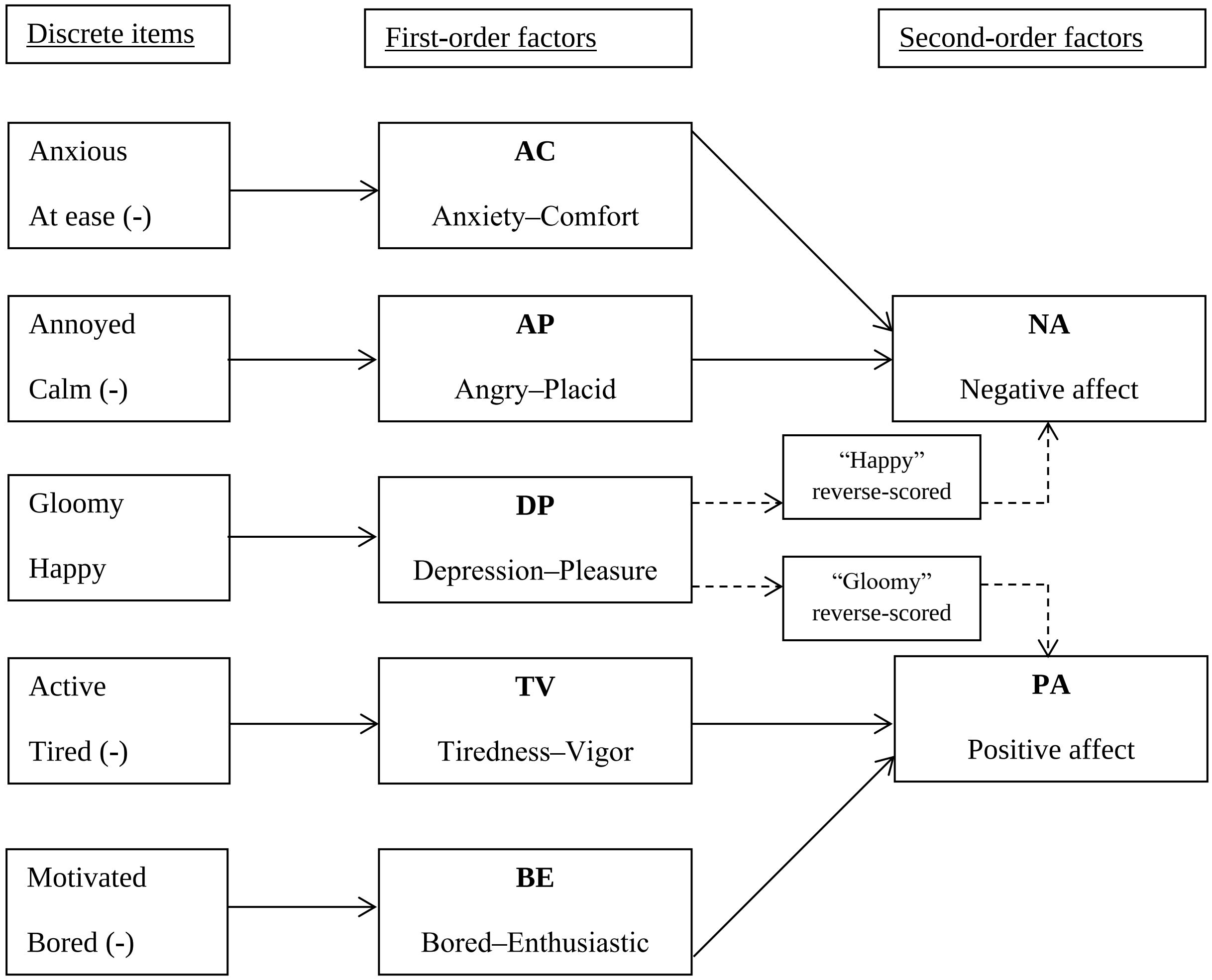

Notes: *without response bias factors; **with response bias factors; NA = negative activated affect; PA = positive activated affect.

Figure 3: The best-fitting factor structure (Model 6) of 10-item Daniels Five-factor measure of Affective Well-being (D-FAW) with unstandardized item loading ranges across the samples (Between/Within Subjects)

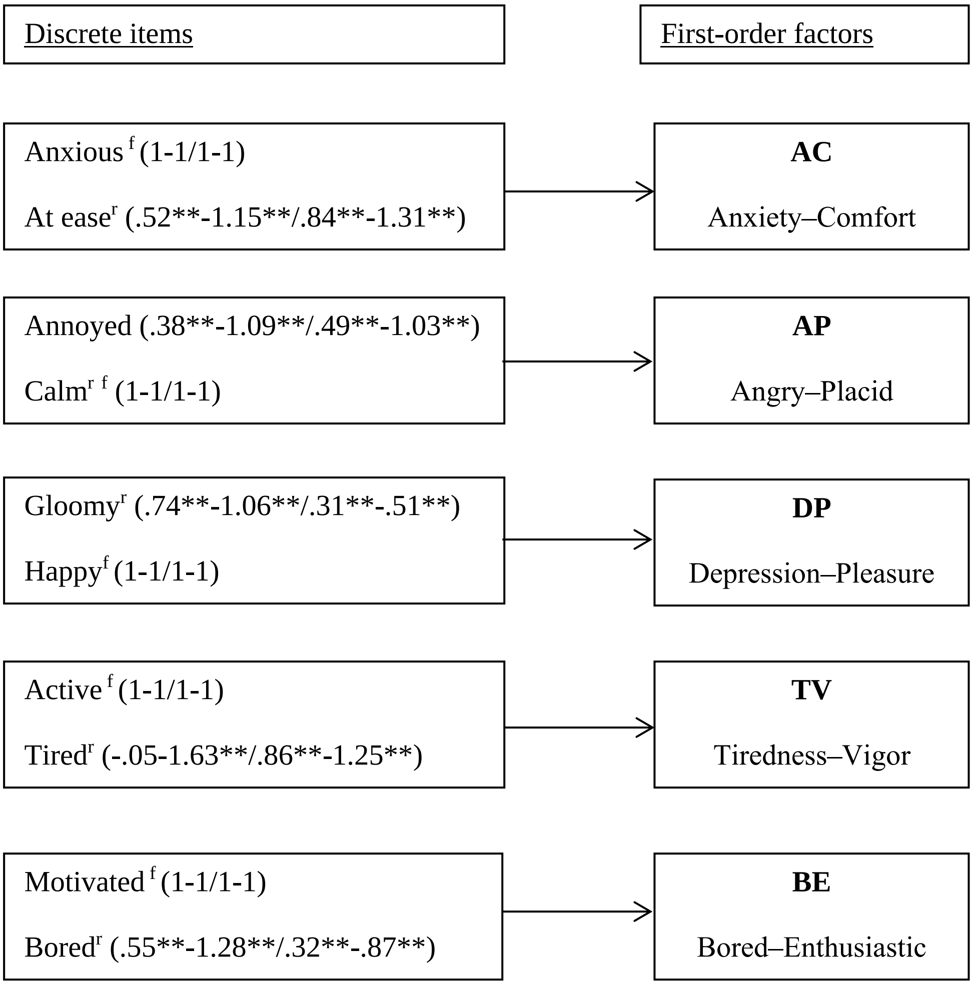

Notes:
[r] Reversed coded
[f] Loading fixed at 1
** p < .05, * p < .01
N.B. Please see Appendix 3 for detailed breakdown of factor loadings per sample at between and within-persons levels across all samples for Model 6. Factor loadings greater than unity are acceptable as these are unstandardized regression weights.

Appendix 2: **The long-form 30-item Daniels Five-factor measure of Affective Well-being (D-FAW) (with 10-items extracted post-administration, highlighted)**

In the section below, please indicate how you feel *right now, that is, at the present moment**. Please circle the most appropriate number on the 6 point scale, where 1 = not at all, to 6 = very much.

Anxious	1	2	3	4	5	6
Worried	1	2	3	4	5	6
Tense	1	2	3	4	5	6
Relaxed	1	2	3	4	5	6
Comfortable	1	2	3	4	5	6
Calm	1	2	3	4	5	6
Depressed	1	2	3	4	5	6
Miserable	1	2	3	4	5	6
Gloomy	1	2	3	4	5	6
Happy	1	2	3	4	5	6
Pleased	1	2	3	4	5	6
Cheerful	1	2	3	4	5	6
Bored	1	2	3	4	5	6
Sluggish	1	2	3	4	5	6
Dull	1	2	3	4	5	6
Enthusiastic	1	2	3	4	5	6
Optimistic	1	2	3	4	5	6
Motivated	1	2	3	4	5	6
Tired	1	2	3	4	5	6
Fatigued	1	2	3	4	5	6
Sleepy	1	2	3	4	5	6
Active	1	2	3	4	5	6
Alert	1	2	3	4	5	6
Full of energy	1	2	3	4	5	6
Angry	1	2	3	4	5	6
Annoyed	1	2	3	4	5	6
Aggressive	1	2	3	4	5	6
Placid	1	2	3	4	5	6
Patient	1	2	3	4	5	6
At ease	1	2	3	4	5	6

Notes: *This focal instruction can be amended according to time frame and context.

Appendix 3: Factor loadings from Model 6 first order model

Scale	Item	Sample 1	Sample 2	Sample 3	Sample 4†	Sample 5†	Sample 6†
AC	At ease	0.81**	1.15**	1.10**	1.03**/0.52**	0.84**/0.64**	1.31**/0.70**
	Anxious[r]	1[f]	1[f]	1[f]	1[f]/1[f]	1[f]/1[f]	1[f]/1[f]
AP	Calm	1[f]	1[f]	1[f]	1[f]/1[f]	1[f]/1[f]	1[f]/1[f]
	Annoyed[r]	0.38**	0.46**	0.38**	0.79**/0.82**	0.49**/1.09**	1.03**/0.45**
DP	Happy	1[f]	1[f]	1[f]	1[f]/1[f]	1[f]/1[f]	1[f]/1[f]
	Gloomy[r]	0.76**	0.81**	0.75*	0.51**/0.74**	0.31**/0.89**	0.37**/1.06**
BE	Motivated	1[f]	1[f]	1[f]	1[f]/1[f]	1[f]/1[f]	1[f]/1[f]
	Bored[r]	1.16**	1.17**	1.28**	0.32**/0.55**	0.62**/0.84**	0.87**/0.74**
TV	Active	1[f]	1[f]	1[f]	1[f]/1[f]	1[f]/1[f]	1[f]/1[f]
	Tired[r]	1.46**	0.12**	-0.05	0.98**/0.39**	0.86**/0.46**	1.25**/1.63**

Notes: AC = Anxiety–Comfort; AP = Angry–Placid; BE = Bored–Enthusiastic; TV = Tiredness–Vigor; DP = Displeasure–Pleasure.
Factor loadings from 1st order model (Model 6)
† Within factor loading / Between factor loading
[r] Reversed coded
[f] Loading fixed at 1
** p < .05, * p < .01

Appendix 4: Bayesian fit and convergence statistics for response bias factors only

	Fit Statistics	Sample 1	Sample 2	Sample 3	Sample 4	Sample 5	Sample 6
Response bias factors only	PSR	= 1.02	= 1.02	= 1.02	= 1.02	= 1.02	= 1.06
	PPC	= <.01	= <.01	= < .01	= <.01	= < .01	= <.01
	DIC	= 7872.86	= 52263.31	= 9017.44	= 7224.85	= 14817.75	= 19109.86
	Loadings	++	++	++	+	++	+

Notes: PSR = potential scale reduction; PPC = posterior predictive checking; DIC = deviance information criteria.
Comparing the results with the results reported in Table 3 reveals that a model with 2 factors representing positively-valenced items only and negatively-valenced items only affect only has less good fit than any model with response bias and substantive factors for any sample.
